# CPEB2 m6A methylation regulates blood–tumor barrier permeability by regulating splicing factor SRSF5 stability

**DOI:** 10.1038/s42003-022-03878-9

**Published:** 2022-09-05

**Authors:** Mengyang Zhang, Chunqing Yang, Xuelei Ruan, Xiaobai Liu, Di Wang, Libo Liu, Lianqi Shao, Ping Wang, Weiwei Dong, Yixue Xue

**Affiliations:** 1 Department of Neurobiology, School of Life Sciences, China Medical University, Shenyang, PR China; 2 Key Laboratory of Cell Biology, Ministry of Public Health of China, China Medical University, Shenyang, PR China; 3 Key Laboratory of Medical Cell Biology, Ministry of Education of China, China Medical University, Shenyang, PR China; 4 Department of Neurosurgery, Shengjing Hospital of China Medical University, Shenyang, PR China; 5 Liaoning Research Center for Translational Medicine in Nervous System Disease, Shenyang, PR China; 6 Key Laboratory of Neuro-oncology in Liaoning Province, Shenyang, PR China

**Keywords:** Epigenetics, CNS cancer

## Abstract

The blood–tumor barrier (BTB) contributes to poor therapeutic efficacy by limiting drug uptake; therefore, elevating BTB permeability is essential for glioma treatment. Here, we prepared astrocyte microvascular endothelial cells (ECs) and glioma microvascular ECs (GECs) as in vitro blood–brain barrier (BBB) and BTB models. Upregulation of METTL3 and IGF2BP3 in GECs increased the stability of *CPEB2* mRNA through its m6A methylation. CPEB2 bound to and increased *SRSF5* mRNA stability, which promoted the ETS1 exon inclusion. P51-ETS1 promoted the expression of ZO-1, occludin, and claudin-5 transcriptionally, thus regulating BTB permeability. Subsequent in vivo knockdown of these molecules in glioblastoma xenograft mice elevated BTB permeability, promoted doxorubicin penetration, and improved glioma-specific chemotherapeutic effects. These results provide a theoretical and experimental basis for epigenetic regulation of the BTB, as well as insight into comprehensive glioma treatment.

## Abstract (web summary)

The methyl transferase METTL3 is up-regulated in brain tumors leading to the methylation of CPEB2 mRNA, which in turn stabilizes the splicing factor SRSF5 mRNA, leading to the incorporation of exon 7 in ETS-1 in models of the Blood–Tumor Barrier.

## Introduction

Glioma is the most common primary tumor of the central nervous system. Currently, the main treatment method is surgery-assisted radiotherapy and chemotherapy. Due to the blood–brain barrier (BBB), it is difficult for macromolecular chemotherapeutics to reach tumor tissues and exert therapeutic effects. The BBB comprises endothelial cells (ECs) with continuous tight junctions and efflux pumps, the basement membrane of the parenchyma (astrocytes), and pericytes. Primary or metastatic tumors in the brain change the BBB to form a reconstructed structure [the blood–tumor barrier (BTB)]^1^. Therefore, selective opening of the BTB is an effective strategy to improve the chemotherapeutic efficacy in brain glioma. Tight junctions comprising tight-junction-related proteins (TJPs) are the main targets for regulating BTB permeability. Downregulation of TJPs, such as the transmembrane proteins occludin, claudin-5, and cytoplasmic plaque protein zonula occludens-1 (ZO-1), can increase BTB permeability^2^. Doxorubicin (Dox), an anthracycline, represses DNA replication, interrupts the cell cycle, and facilitates the generation of intracellular reactive oxygen species to induce tumor cell death^3–6^. Dox is unable to penetrate the BBB by itself but exhibits potent cytotoxicity when cultured with glioma cells^7,8^. In the present study, we examined the leakage of Dox in glioblastoma multiforme (GBM) orthotopic xenograft nude mice, as well as the size of the grafted tumors in nude mice, to evaluate BTB permeability.

N6-methyladenosine (m6A), which mainly occurs on the adenine of the highly conserved RRACH (R: purine; A: m6A; H: nonguanine) sequence, is the most abundant form of methylation modification in eukaryotic mRNAs, and its function is determined by the methyltransferase (encoder), demethylase (decoder), and binding protein (code reader)^9^. Increasing studies have shown that m6A can functionally regulate eukaryotic transcriptome functions, such as mRNA splicing, nucleation, localization, translation, and stabilization^10^. Moreover, m6A can determine the fate of hematopoietic stem cells during vertebrate embryo development^11^. Methyltransferase 3 (METTL3), among the first identified m6A methyltransferases, is highly expressed in a variety of tumor tissues, where it promotes mRNA translation and regulates tumor cell proliferation by regulation of the methylation of target genes. Insulin-like growth factor 2 mRNA-binding protein 3 (IGF2BP3), a member of the m6A-reading family of IGF2BPs, enhances mRNA stability and translation by recognizing the GG (m^6^A) C sequence shared by mRNAs^12^. Interestingly, IGF2BP3 is highly expressed in lung and breast cancers, where it regulates tumor occurrence and development^13^. However, few studies have focused on the roles of METTL3 and IGF2BP3 in the regulation of the vascular endothelial cell.

In this study, we revealed mechanisms associated with METTL3-mediated m6A modification and its role in regulating BTB permeability. Moreover, we identified methods for selectively opening the BTB, increasing drug penetration into tumor tissues, and improving chemotherapeutic efficacy.

## Results

### Knockdown of Upregulated METTL3 and IGF2BP3 Levels in GECs Increases BTB Permeability

**Fig. 1:**
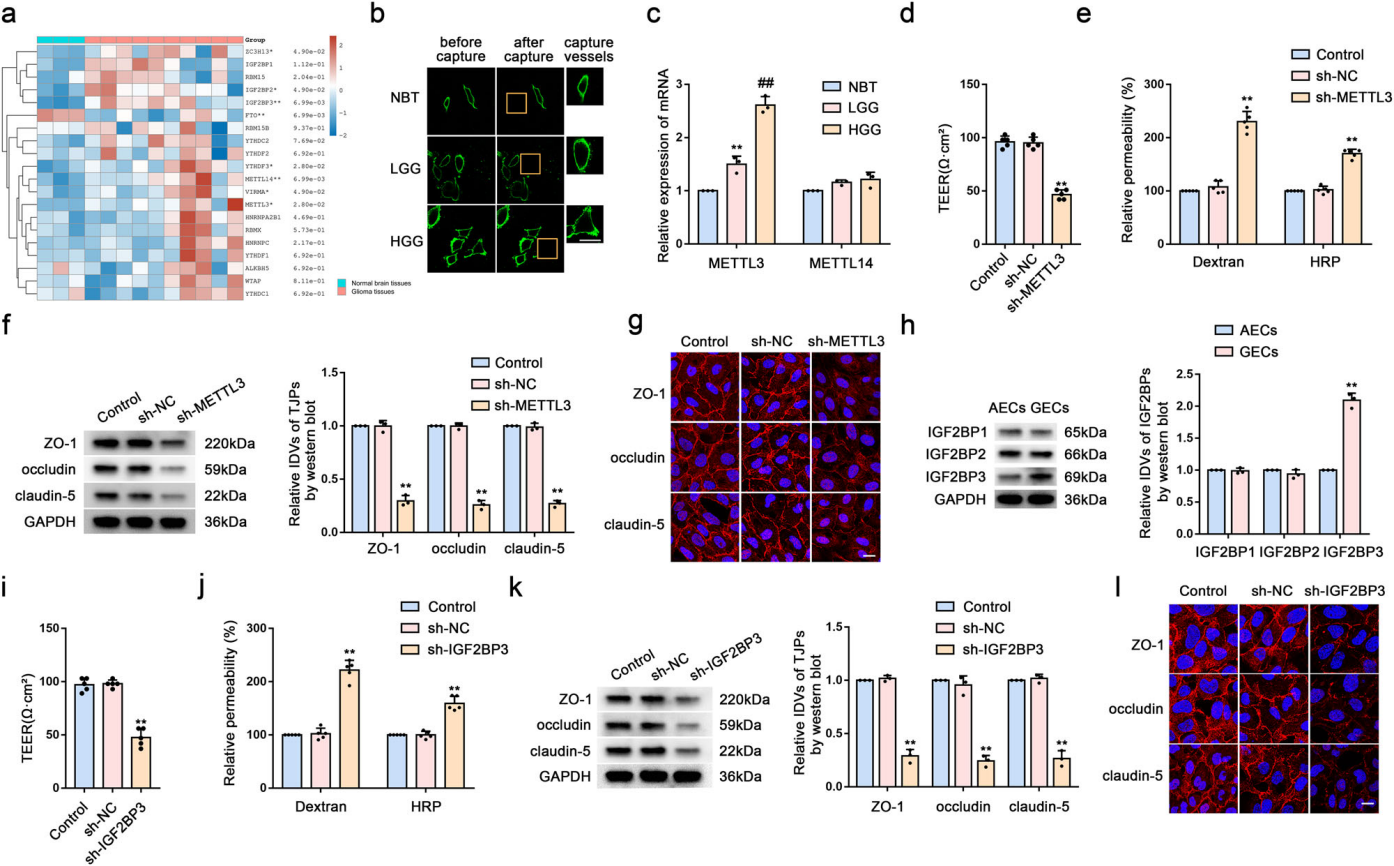
Knockdown of Upregulated METTL3 and IGF2BP3 Levels in GECs Increases BTB Permeability. **a** m6A related gene expression heat map analyzed using GEO database, where different colors represent the expression trend in different samples. ^*^P  <  0.05, ^**^P  <  0.01, ^***^P  <  0.001, the asterisk represents the degree of importance (^*^P). The significance of the two groups of samples passed the Wilcox test, and the significance of the three groups and above passed the Kruskal-Wallis test. **b** Staining vessels from glioma or normal brain tissues with UEA-I. LCM capture of UEA-I-stained vessel from human brain sections is shown. Scale bar represents 40 µm. **c** qRT-PCR analysis of *METTL3 and METTL14* expression in brain microvessels from NBT, LGG, and HGG by LCM. Data are represented as mean ± SD (*n* = 3). ^**^P < 0.01 vs. NBT group; ^##^*P* < 0.01 vs. LGG group. **d**, **e** The permeability and integrity of the METTL3 knockdown in vitro BTB model were detected by TEER values, FITC-dextran, and HRP flux. Data are represented as mean ± SD (*n* = 5). ^**^P < 0.01 vs. sh-NC group. **f** The expressions of ZO-1, occludin, and claudin-5 in the METTL3 knockdown GECs detected by western blot analysis (IDVs, integrated densitometry values). Data were represented as mean ± SD (*n* = 3). ^**^P < 0.01 vs. sh-NC (NC, negative control) group. **g** The distributions of ZO-1, occludin and claudin-5 in the METTL3 knockdown GECs were observed by IF staining. The scale bar represents 50 µm. **h** Relative protein levels of IGF2BP1, IGF2BP2, and IGF2BP3 in AECs and GECs were determined by western blot analysis. Data are represented as mean ± SD (*n* = 3). ^**^P < 0.01 vs. AECs group. **i**, **j** The permeability and integrity of the IGF2BP3 knockdown BTB model in vitro were detected by TEER values, FITC-dextran, and HRP flux. Data are represented as mean ± SD (*n* = 5). ^**^P < 0.01 vs. sh-NC group. **k** The expressions of ZO-1, occludin, and claudin-5 in the IGF2BP3 knockdown GECs were detected by western blot assays. Data are represented as mean ± SD (*n* = 3). ^**^P < 0.01 vs. sh-NC group. **l** The distributions of ZO-1, occludin and claudin-5 in the IGF2BP3 knockdown GECs were determined by IF staining. Scale bar represents 50 µm.

We first analyzed the differentially expressed m6A-related genes in glioma and non-tumor brain tissue using the Gene Expression Omnibus (GEO) database (https://www.ncbi.nlm.nih.gov/geo/) (Figs. 1a and S1a). To evaluate the BTB at the molecular level, we used LCM to capture brain microvessels in NBT, LGG, and HGG from resected human brain specimens (Fig. 1b) and determining levels of METTL3 and METTL14 mRNA using qRT-PCR. We found that compared with that in NBT, METTL3 mRNA level increased significantly in LGG and HGG microvessels, whereas METTL14 mRNA level showed no significant difference (Fig. 1c). We then co-cultured three cell lines (U87, U373, and U251) with ECs to establish in vitro BTB models (GECs), with NHAs co-cultured with ECs (AECs) used as controls. Western blot analysis showed that METTL3 levels in GECs from the co-culture of ECs with U251 increased significantly relative to those in controls (Fig. S1b); therefore, we used GECs from co-cultured ECs and U251 for subsequent experiments. Figure S1c shows a simple schematic of the in vitro BBB and BTB models. We then determined the TEER value and used FITC–dextran tracers and HRP flux to evaluate BBB and BTB permeability, with the results revealing BTB permeability as higher than that of the BBB (Fig. S1d, e). We then knocked down METTL3 levels in GECs using shRNA [sh-METTL3 and sh-negative control (sh-NC)] and measured the TEER value, FITC-dextran, and HRP flux to analyze BBB integrity and permeability. We found that compared with the sh-NC group (cells transfected with empty plasmids), the TEER value of the sh-METTL3 group was significantly reduced, whereas FITC–dextran and HRP-flux signals increased significantly (Fig. 1d, e). Additionally, western blot analysis indicated significant decreases in ZO-1, occludin, and claudin-5 levels in the sh-METTL3 group (Fig. 1f). Moreover, IF assays revealed the continuous distribution of TJPs in the control and sh-NC groups, whereas this was not the case in the sh-METTL3 group (Fig. 1g).

As shown in Figs. 1a and S1a, we noted significantly higher expression of the m6A-binding protein IGF2BP in GBM tissues relative to NBT. Therefore, upon investigating the expression levels of IGF2BP1, IGF2BP2, and IGF2BP3 in AECs and GECs, we found IGF2BP3 expression levels to be significantly elevated in GECs relative to those in AECs (Fig. 1h). Notably, after IGF2BP3 knockdown, the TEER values, expression levels, and continuous distribution of ZO-1, occludin, and claudin-5 decreased significantly, whereas the FITC–dextran and HRP-flux signals increased significantly, relative to observations in the sh-NC group (Fig. 1i–l).

### Knockdown of elevated CPEB2 expression in GECs increases BTB permeability

**Fig. 2:**
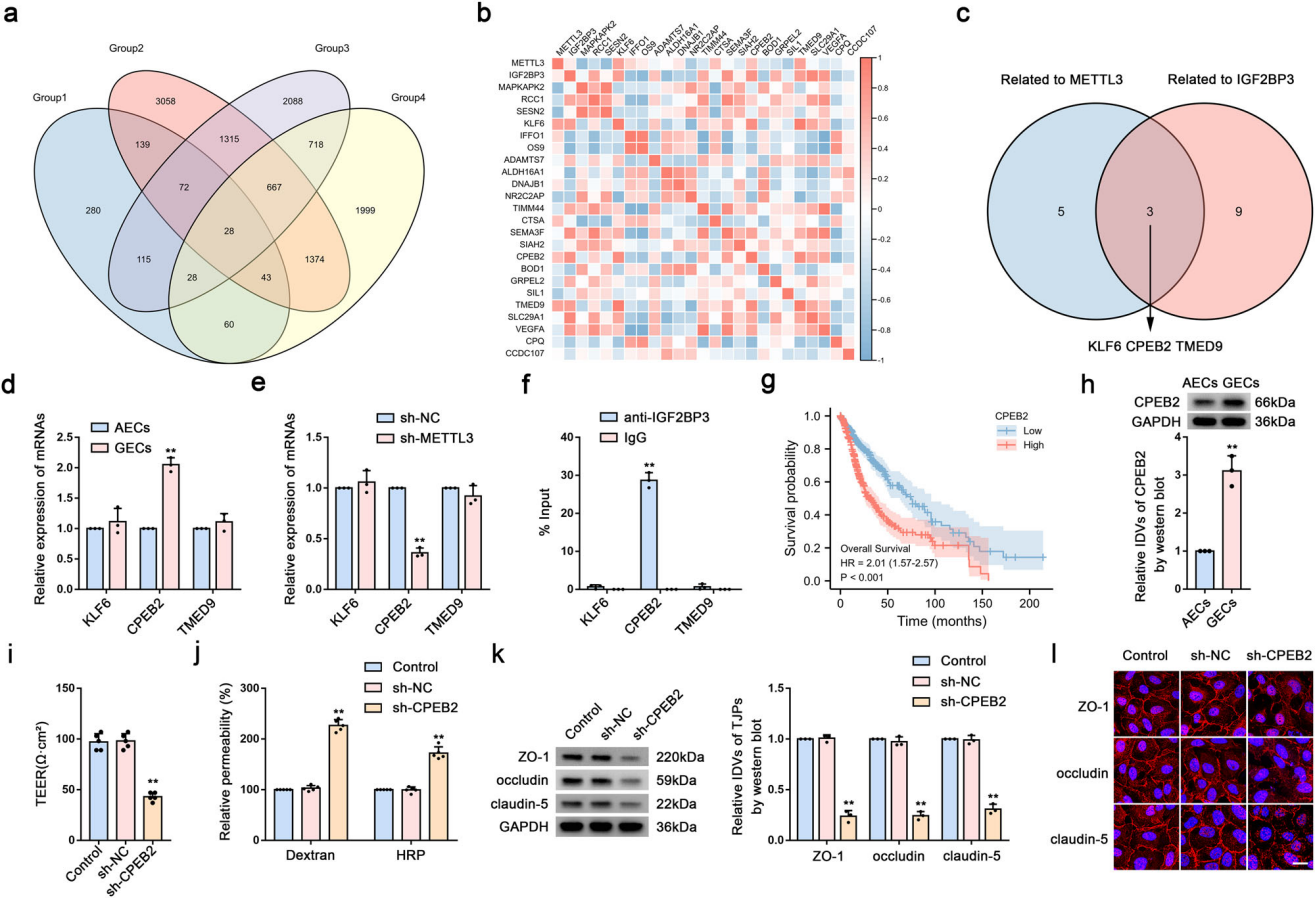
Knockdown of elevated CPEB2 Expression in GECs Increases BTB Permeability. **a** Venn diagram. (Group1, MeRIP-seq of scramble and METTL3 knockdown HEK-293T cell, GSE182607; Group2, Transcriptome analysis of gene expression in scramble and METTL3 knockdown HUVEC (human umbilical vein endothelial cells) cell lines, GSE157544; Group 3, RNA deep sequencing analysis of human brain microvascular endothelial cells (ECs) treated with glioma-conditioned medium (glioma-CM), GSE115850; Group 4, Genes positively associated with poor prognosis in glioma.) **b** Correlation heatmap of METTL3 and IGF2BP3 with differentially expressed factors in glioma, analyzed using TCGA database. **c** Venn diagram. **d** Relative mRNA levels of *KLF6, CPEB2, and TMED9* in AECs and GECs were determined by qRT-PCR. Data are represented as mean ± SD (*n* = 3). ^**^P < 0.01 vs. AECs group. **e** Relative mRNA levels of *KLF6, CPEB2, and TMED9* in sh-METTL3 GECs were determined by qRT-PCR. Data are represented as mean ± SD (*n* = 3). ^**^P < 0.01 vs. sh-NC group. **f** RIP was performed with normal mouse IgG or anti-IGF2BP3 antibody in GECs. Data are represented as mean ± SD (*n* = 3). ^**^P < 0.01 vs. anti-IgG group. Relative enrichment of KLF6, CPEB2, and TMED9 was determined by qRT-PCR. **g** CPEB2 prognostic analysis in glioma using TCGA database. **h** Relative protein levels of CPEB2 in AECs and GECs were determined by western blot analysis. **i**, **j** The permeability and integrity of the CPEB2 knockdown in vitro BTB model were detected by TEER values, FITC-dextran, and HRP flux. Data are represented as mean ± SD (*n* = 5). ^**^P < 0.01 vs. sh-NC group. **k** The expressions of ZO-1, occludin, and claudin-5 in the CPEB2 knockdown GECs detected by western blot analysis. Data are represented as mean ± SD (*n* = 3). ^**^P < 0.01 vs. sh-NC group. **l** The distributions of ZO-1, occludin, and claudin-5 in the CPEB2 knockdown GECs were observed by IF staining. Scale bar represents 50 µm.

We analyzed the RNA-Seq and MeRIP-Seq data from the GSE115850, GSE157544, and GSE182607 datasets from the GEO database and TCGA for molecules associated with glioma prognosis. We detected 28 factors that were differentially expressed in the sh-METTL3 group (Fig. 2a), with correlation analysis of the expression profiles of these factors in ECs performed using the BBBomics database (http://bioinformaticstools.mayo.edu/bbbomics/). The correlation heatmap shown in Fig. 2b revealed correlations of three molecules with METTL3 and IGF2BP3 (Fig. 2c). We then detected mRNA levels in AECs and GECs, finding that *CPEB2* mRNA level was significantly elevated in GECs (Fig. 2d) and significantly decreased in the sh-METTL3 group (Fig. 2e). Additionally, RIP experiments showed that *CPEB2* mRNA level was significantly enriched by the introduction of the IGF2BP3 antibody (Fig. 2f). Further analysis of TCGA data indicated that CPEB2 was significantly associated with poor prognosis in glioma (Fig. 2g). Moreover, we observed that CPEB2 protein level significantly higher in GECs than in AECs (Fig. 2h), as well as that the sh-CPEB2 group exhibited a significant decrease in the TEER value and significant increases in FITC–dextran and HRP-flux signals relative to the sh-NC group (Fig. 2i, j). Furthermore, western blotting and IF assays confirmed significant reductions in ZO-1, occludin, and claudin-5 levels in the sh-CPEB2 group relative to levels in the sh-NC group (Fig. 2k, l).

### METTL3-mediated m6A modification maintains CPEB2 levels via IGF2BP3-dependent regulation of *CPEB2* mRNA stability and is essential for regulating BTB permeability

**Fig. 3:**
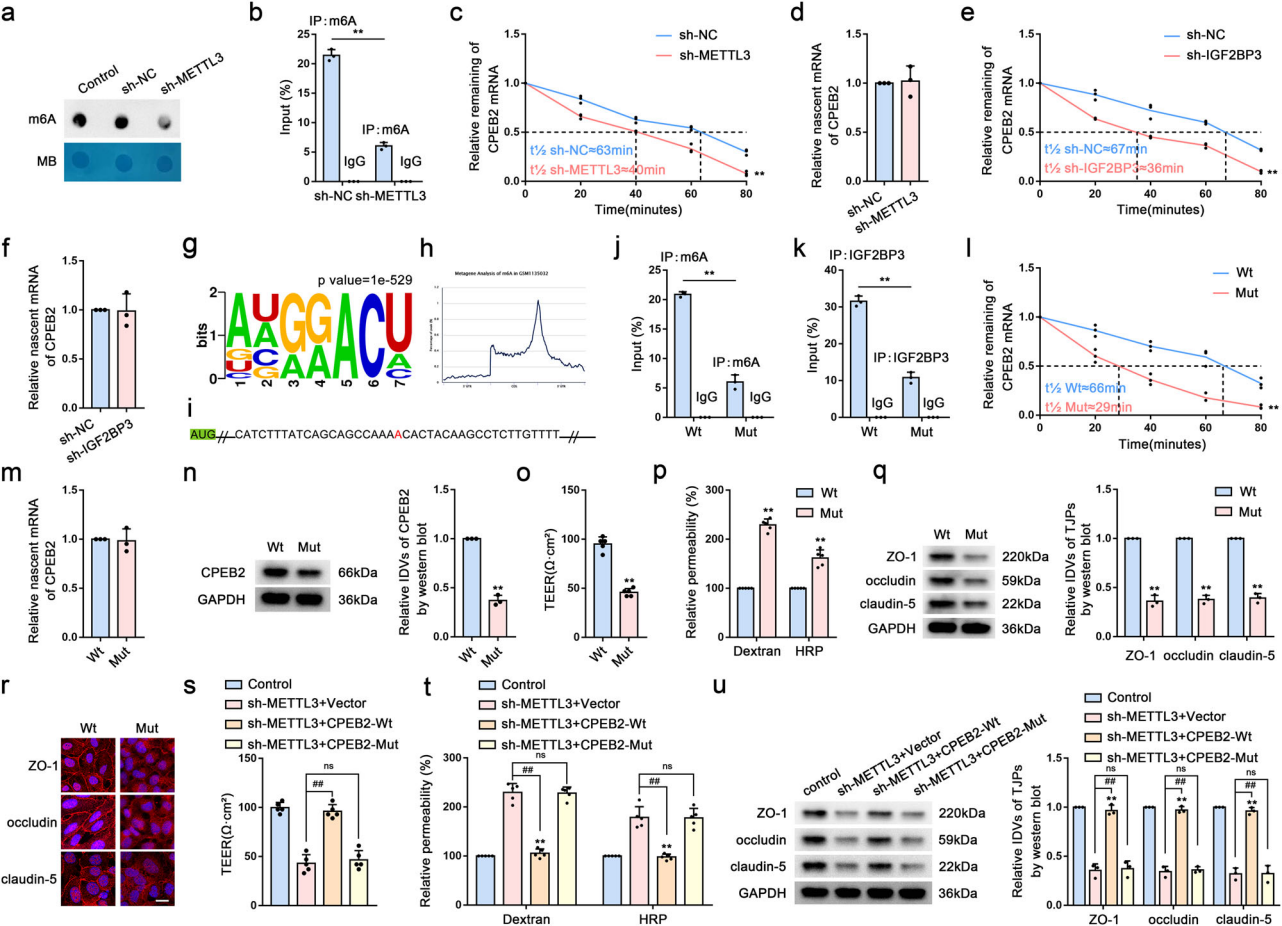
METTL3-mediated m6A Modification Maintains CPEB2 Levels via IGF2BP3-dependent regulation of *CPEB2* mRNA stability and is essential for Regulating BTB Permeability. **a** M6A dot blot assays of METTL3 knockdown GECs. Methylene blue staining served as a loading control. **b** Relative enrichment of CPEB2 m6A modification in the METTL3 knockdown GECs vs. sh-NC group were analyzed using qRT-PCR. **c** Relative expression levels of *CPEB2* mRNA in the METTL3 knockdown GECs treated with actinomycin D at different time points were analyzed using qRT-PCR. **d** Relative expression levels of nascent CPEB2 in the METTL3 knockdown GECs were detected using qRT-PCR. **e** Relative expression levels of *CPEB2* mRNA in the IGF2BP3 knockdown GECs treated with actinomycin D at different time points were analyzed using qRT-PCR. **f** Relative expression levels of nascent CPEB2 in the IGF2BP3 knockdown GECs were detected using qRT-PCR. Data are represented as mean ± SD (*n* = 3). **P < 0.01 vs. sh-NC group. **g** M6A motif visualization. **h** Metagene analysis of m6A in GSM1135032. **i** CPEB2 m6A mutation site. **j** Relative enrichment of CPEB2 m6A modification in Mut group vs. Wt group were analyzed using qRT-PCR. **k** RIP was performed with normal mouse IgG or anti-IGF2BP3 antibody in Mut group vs. Wt group. **l** Relative expression levels of *CPEB2* mRNA in Mut group treated with actinomycin D at different time points were analyzed using qRT-PCR. **m** Relative expression levels of nascent CPEB2 in Mut group were detected using qRT-PCR. **n** Relative protein levels of CPEB2 in Wt and Mut group were determined by western blot analysis. **o**, **p** TEER values, FITC-dextran, and HRP flux of the Wt and Mut group. Data are represented as mean ± SD (*n* = 5). ^**^P < 0.01 vs. Wt group. **q** The expressions of ZO-1, occludin, and claudin-5 in the Wt and Mut group detected by western blot analysis. Data were represented as mean ± SD (*n* = 3). ^**^P < 0.01 vs. Wt group. **r** The distributions of ZO-1, occludin, and claudin-5 in the Wt and Mut group were determined by IF staining. Scale bar represents 50 µm. **s**, **t** The permeability and integrity of sh-METTL3 + CPEB2-Wt and sh-METTL3 + CPEB2-Mut BTB model in vitro were detected by TEER values, FITC-dextran and HRP flux. Data are represented as mean ± SD (*n* = 5). ^**^P < 0.01 vs. sh-METTL3 + Vector group. **u** The expressions of ZO-1, occludin, and claudin-5 in sh-METTL3 + CPEB2-Wt and sh-METTL3 + CPEB2-Mut BTB model detected by western blot assays. Data are represented as mean ± SD (*n* = 3). ^**^P < 0.01 vs. sh-METTL3 + Vector group.

To determine the molecular mechanism underlying inhibition of BTB permeability by METTL3, we measured the overall m6A level in the sh-NC and sh-METTL3 groups using m6A dot blotting. We found significantly reduced m6A levels in the sh-METTL3 group (Fig. 3a). To determine whether the m6A modification of CPEB2 is mediated by METTL3, we performed MeRIP-qPCR analysis in sh-METTL3 GECs, revealing significant reductions in m6A-modified CPEB2 mRNA following METTL3 knockdown (Fig. 3b). Subsequently, we used mRNA half-life and nascent RNA assays to investigate the effect of METTL3 and IGF2BP3 on *CPEB2* mRNA stability. Although the half-life of *CPEB2* mRNA was significantly shorter following METTL3 knockdown (Fig. 3c), we detect no significant change in nascent *CPEB2* mRNA levels in the sh-METTL3 group relative to the sh-NC group (Fig. 3d), with similar results obtained in the sh-IGF2BP3 group (Fig. 3e, f). Using the RMBase database (https://rna.sysu.edu.cn/rmbase/index.php), we found that m6A modification occurs in *CPEB2* mRNA in the 3′ untranslated region (UTR) (Fig. 3g, h). To determine whether CPEB2 modifies BTB permeability according to the levels of m6A modification to *CPEB2* mRNA, we mutated the m6A-methylation site and stably transfected GECs with either wild-type (Wt) or mutant (Mut) constructs (Fig. 3i). The results showed significant reductions in methylation levels in the Mut group according to MeRIP-qPCR analysis (Fig. 3j). Additionally, RIP assay revealed that significantly lower levels of CPEB2 enrichment by the IGP2BP3 antibody in the Mut group relative to than in the Wt group (Fig. 3k). Notably, we observed that *CPEB2* mRNA stability was significantly reduced in the Mut group, whereas nascent RNA levels did not change (Fig. 3l, m). Moreover, CPEB2 protein level in the Mut group was significantly lower than that in the Wt group (Fig. 3n), and in the Mut group, the TEER value and levels of ZO-1, occludin, and claudin-5 were also significantly reduced, whereas the FITC–dextran and HRP-flux signals and BTB permeability increased significantly (Fig. 3o–r). We subsequently overexpressed CPEB2-Wt and CPEB2-Mut in sh-METTL3 GECs, with the results showing that CPEB2-Wt overexpression significantly reversed the effects of sh-METTL3 on the TEER value, HRP flux, and the status of TJPs, whereas CPEB2-Mut overexpression had no effect on these parameters (Fig. 3s–u).

### CPEB2 enhances *SRSF5* mRNA stability and regulates BTB permeability

**Fig. 4:**
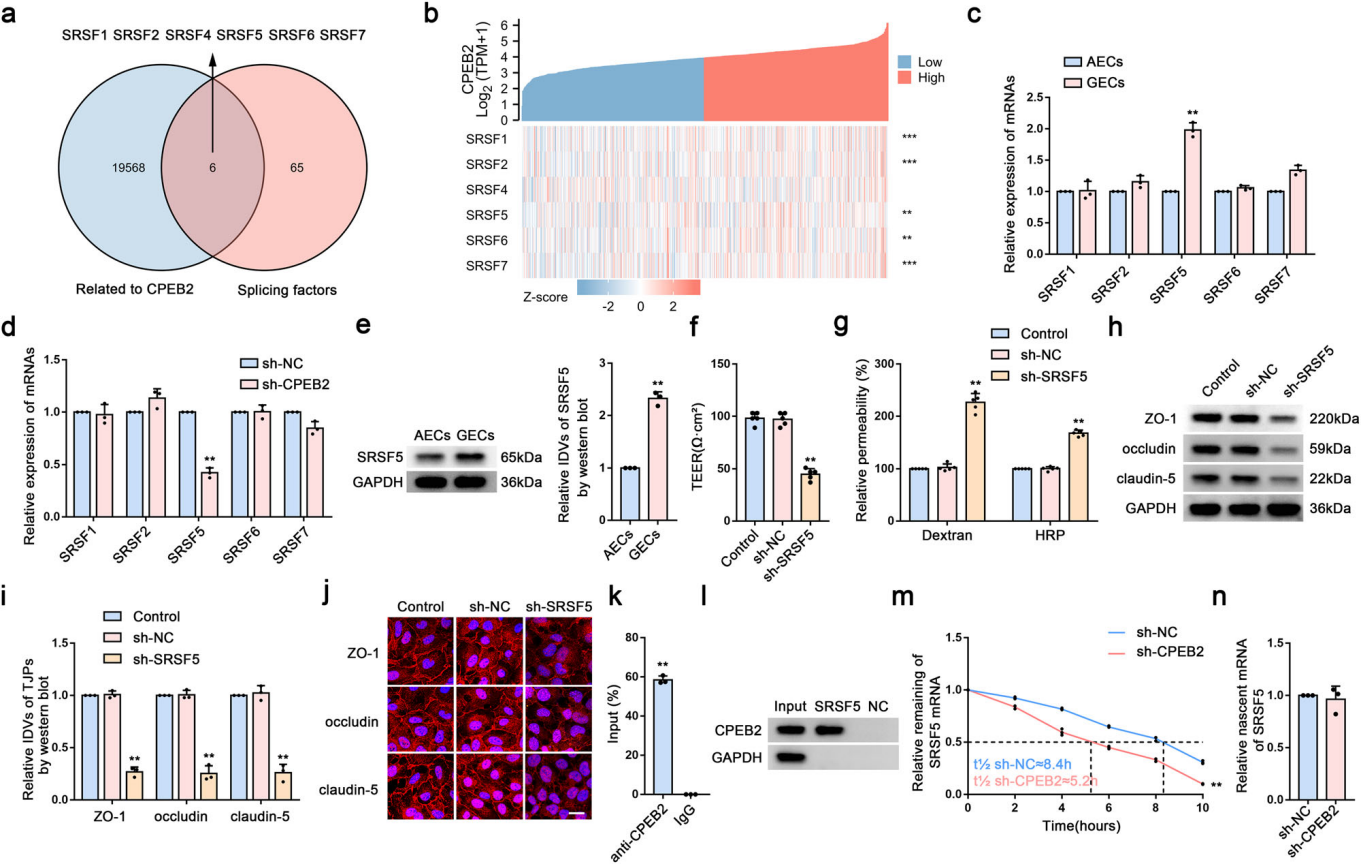
CPEB2 Enhances *SRSF5* mRNA Stability and Regulates BTB Permeability. **a** Venn diagram for screening SRSF5 using datasets. **b** Single-gene co-expression analysis of CPEB2 with these molecules in glioma using data from the TCGA database. **c** Relative mRNA levels of SRSFs in AECs and GECs were determined by qRT-PCR. **d** Relative mRNA levels of SRSFs in sh-CPEB2 GECs were determined by qRT-PCR. **e** Relative protein levels of SRSF5 in AECs and GECs were determined by western blot analysis. Data were represented as mean ± SD (*n* = 3). ^**^P < 0.01 vs. AECs group. **f**, **g** The permeability and integrity of the SRSF5 knockdown in vitro BTB model were detected by TEER values, FITC-dextran, and HRP flux. Data are represented as mean ± SD (*n* = 5). ^**^P < 0.01 vs. sh-NC group. **h**, **i** The expressions of ZO-1, occludin and claudin-5 in the SRSF5 knockdown GECs were detected by western blot analysis. Data are represented as mean ± SD (*n* = 3). ^**^P < 0.01 vs. sh-NC group. **j** The distributions of ZO-1, occludin and claudin-5 in the SRSF5 knockdown GECs were determined by IF staining. Scale bar represents 50 µm. **k** Relative enrichment of SRSF5 was determined by qRT-PCR. Data are represented as mean ± SD (*n* = 3). ^**^P < 0.01 vs. anti-IgG group. **l** CPEB2 and GAPDH protein levels in immunoprecipitation with *SRSF5* mRNA were evaluated by western blot analysis. The expression levels of CPEB2 and GAPDH proteins are shown. **m** Relative expression levels of *SRSF5* mRNA in the CPEB2 knockdown GECs treated with actinomycin D at different time points were analyzed using qRT-PCR. **n** Relative expression levels of nascent *SRSF5* mRNA in the CPEB2 knockdown GECs were detected using qRT-PCR. Data are represented as mean ± SD (*n* = 3). ^**^P < 0.01 vs. sh-NC group.

We then performed a single-gene differential analysis of CPEB2 through TCGA and analyzed the splicing factors retrieved from the SpliceAid 2 database (http://193.206.120.249/splicing_tissue). The Venn diagram showed six splicing factors in the intersection (Fig. 4a). Single-gene co-expression analysis of *CPEB2* with these molecules identified *SRSF1, SRSF2, SRSF5, SRSF6*, and *SRSF7* as showing a significant positive correlation with *CPEB2* expression (Fig. 4b). We further examined their respective mRNA levels in AECs and GECs, finding that *SRSF5* mRNA level was significantly elevated in GECs (Fig. 4c). Subsequent detection of *SRSF5* mRNA level in GECs revealed a significant decrease in the sh-CPEB2 group (Fig. 4d), with confirmation that SRSF5 protein level was significantly higher in GECs than in AECs (Fig. 4e). Additionally, compared with the sh-NC group, the TEER value and protein levels of ZO-1, occludin, and claudin-5 were significantly reduced, whereas FITC–dextran and HRP-flux signals increased significantly in the sh-SRSF5 group (Fig. 4f–i). Moreover, these proteins were discontinuously distributed in the sh-SRSF5 group according to IF staining (Fig. 4j). Furthermore, RIP and RNA pulldown analyses revealed the ability of CPEB2 to bind to *SRSF5* mRNA (Fig. 4k, l). Further investigation of the mechanism associated with SRSF5 regulation by CPEB2 using sh-CPEB2 GECs and RNA-stability and nascent RNA assays revealed that the half-life of *SRSF5* mRNA was significantly shorter in sh-CPEB2 GECs relative to that in sh-NC GECs (Fig. 4m), whereas the nascent *SRSF5* mRNA level remained unchanged (Fig. 4n).

### CPEB2 regulates BTB permeability via SRSF5

**Fig. 5:**
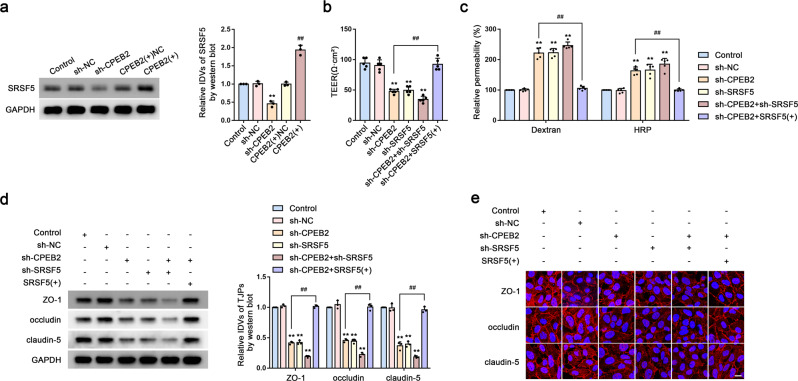
CPEB2 Regulates BTB Permeability via SRSF5. **a** The expressions of SRSF5 in sh-CPEB2 and CPEB2( + ) BTB model were detected by western blot analysis. Data are represented as mean ± SD (*n* = 3). ^**^P < 0.01 vs. sh-NC group, ^##^*P* < 0.01 vs. CPEB2( + ) NC group. **b**, **c** The permeability and integrity of the sh-CPEB2, sh-SRSF5, sh-CPEB2 + sh-SRSF5, sh-CPEB2 + SRSF5( + ) BTB model in vitro were detected by TEER values, FITC-dextran, and HRP flux. Data are represented as mean ± SD (*n* = 5). ^**^P < 0.01 vs. sh-NC group, ^##^*P* < 0.01 vs. sh-CPEB2 group. **d** The expressions of ZO-1, occludin, and claudin-5 in sh-CPEB2, sh-SRSF5, sh-CPEB2 + sh-SRSF5, sh-CPEB2 + SRSF5( + ) BTB model detected by western blot analysis. Data are represented as mean ± SD (*n* = 3). ^**^P < 0.01 vs. sh-NC group, ^##^*P* < 0.01 vs. sh-CPEB2 group. **e** The distributions of ZO-1, occludin and claudin-5 in sh-CPEB2, sh-SRSF5, sh-CPEB2 + sh-SRSF5, sh-CPEB2 + SRSF5(+) BTB model were determined by IF staining. Scale bar represents 50 µm.

We then performed western blot analysis to detect protein levels of SRSF5 in CPEB2-knockdown and -overexpressing GECs, with the results showing significant decreases and increases in SRSF5 expression in these groups, respectively (Fig. 5a). Additionally, we showed that the TEER value and TJP levels were significantly decreased, whereas FITC–dextran and HRP-flux signals increased significantly in the sh-CPEB2, sh-SRSF5, and sh-CPEB2 + sh-SRSF5 groups. However, in the sh-CPEB2 + SRSF5-overexpression [SRSF5( + )] group, we observed a reversal of the effects caused by CPEB2 knockdown (Fig. 5b–e).

### SRSF5 induces ETS1 exon 7 inclusion to regulate BTB permeability

**Fig. 6:**
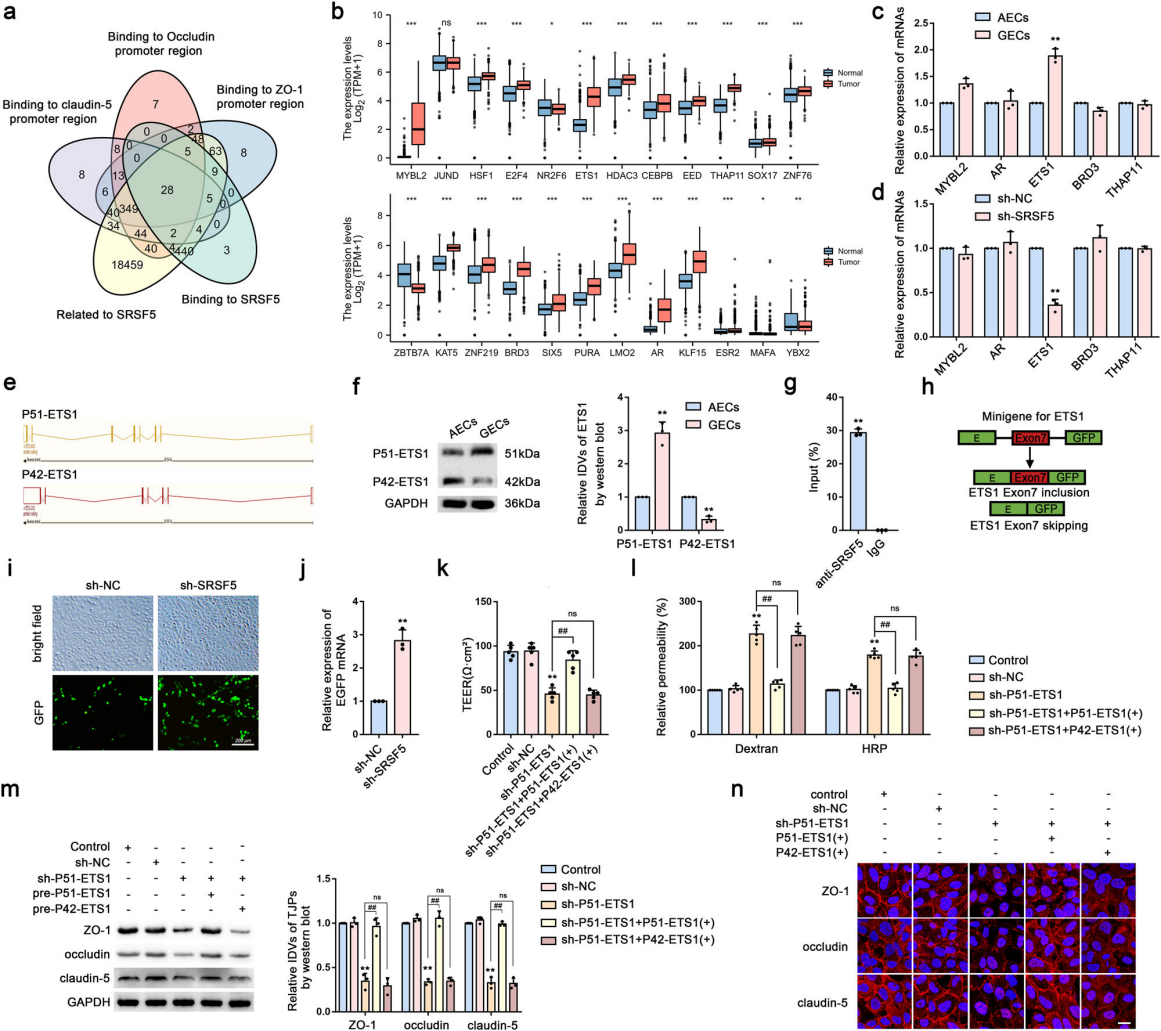
SRSF5 Induces ETS1 Exon 7 Inclusion to Regulate BTB Permeability. **a** Venn diagram. **b** Expression of differentially factors in normal brain tissues and glioma was analyzed by the GTEx and TCGA databases. **c** Relative mRNA levels of top5 high expression genes in AECs and GECs were determined by qRT-PCR. Data are represented as mean ± SD (*n* = 3). ^**^P < 0.01 vs. AECs group. **d** Relative mRNA levels of the top5 high expression genes in sh-SRSF5 GECs were determined by qRT-PCR. Data are represented as mean ± SD (*n* = 3). ^**^P < 0.01 vs. sh-NC group. **e** Spliceosomes of ETS1 (P51-ETS1 and P42-ETS1). **f** Relative protein levels of P51-ETS1 and P42-ETS1 in AECs and GECs were determined by western blot analysis. Data are represented as mean ± SD (*n* = 3). ^**^P < 0.01 vs. AECs group. **g** RIP for detecting the interaction between SRSF5 protein and *ETS1* mRNA. **h** Minigene reporter system for detecting ETS1 exon7 splicing. **i** The fluorescence signal in the GFP channel represents exon7 splicing efficiency in sh-NC and sh-SRSF5 GECs. Scale bar represents 200 µm. **j** Quantification of splicing efficiency by measuring the relative expression of intact EGFP transcript *ETS1* mRNA levels. **k**, **l** TEER values, FITC-dextran and HRP flux of sh-P51-ETS1, sh-P51-ETS1 + P51-ETS1( + ), sh-P51-ETS1 + P42-ETS1( + ) group. Data are represented as mean ± SD (*n* = 5). ^**^P < 0.01 vs. sh-NC group, ^##^*P* < 0.01 vs. sh-P51-ETS1 group. **m** Effects of sh-P51-ETS1, sh-P51-ETS1 + P51-ETS1( + ), sh-P51-ETS1 + P42-ETS1( + ) group on the expressions of ZO-1, occludin and claudin-5 were analyzed by western blot assays. Data are represented as mean ± SD (*n* = 3). ^**^P < 0.01 vs. sh-NC group, ^##^*P* < 0.01 vs. sh-P51-ETS1 group. **n** The distributions of ZO-1, occludin and claudin-5 in the sh-P51-ETS1, sh-P51-ETS1 + P51-ETS1( + ), sh-P51-ETS1 + P42-ETS1( + ) GECs were determined by IF staining. The scale bar represents 50 µm.

We predicted the transcription factors that bind to the promoter regions of ZO-1, occludin, and claudin-5, respectively, using the Jaspar database (https://jaspar.genereg.net/) and predicted SRSF5-specific RNA-binding motifs using the catRAPID database (http://service.tartaglialab.com/page/catrapid_group). Additionally, we used TCGA to identify co-interaction networks involving SRSF5 (Fig. 6a) and GTEx and TCGA to evaluate the expression of these factors in NBT and glioma tissues (Fig. 6b), with the five molecules showing the highest expression selected for qRT-PCR verification. We found that *ETS1* mRNA level was significantly increased in GECs (Fig. 6c), and that these levels were significantly decreased following SRSF5 knockdown (Fig. 6d). Interestingly, ETS1 contains multiple splice variants (Fig. 6e), and we found that levels of the P51-ETS1 protein were significantly higher in GECs than in AECs, whereas P42-ETS1 level was significantly lower in GECs than in AECs (Fig. 6f). RIP assays subsequently confirmed that SRSF5 binds to *ETS1* mRNA (Fig. 6g). We then performed *ETS1* exon 7 minigene assays. Figure 6h shows that expression of EGFP signaled the retention of the entire open reading frame (ORF) after skipping exon 7 (resulting in translation of P42-ETS1), whereas inclusion of exon 7 (resulting in translation of P51-ETS1) altered the ORF sequence and reduced EGFP expression. Notably, we observed a significant increase in EGFP expression following SRSF5 knockdown, indicating that SRSF5 promotes the retention of *ETS1* exon 7 (Fig. 6i, j). We then overexpressed P51-ETS1 and P42-ETS1 separately in sh-P51-ETS1 GECs, finding that P51-ETS1 overexpression in the sh-P51-ETS1 [sh-P51-ETS1 + P51-ETS1( + )] group reversed the effects of sh-P51-ETS1, whereas no change was observed in the sh-P51-ETS1 + P42-ETS1( + ) group (Fig. 6k–n).

### P51-ETS1 regulates BTB permeability by promoting the transcription of ZO-1, occludin, and claudin-5

**Fig. 7:**
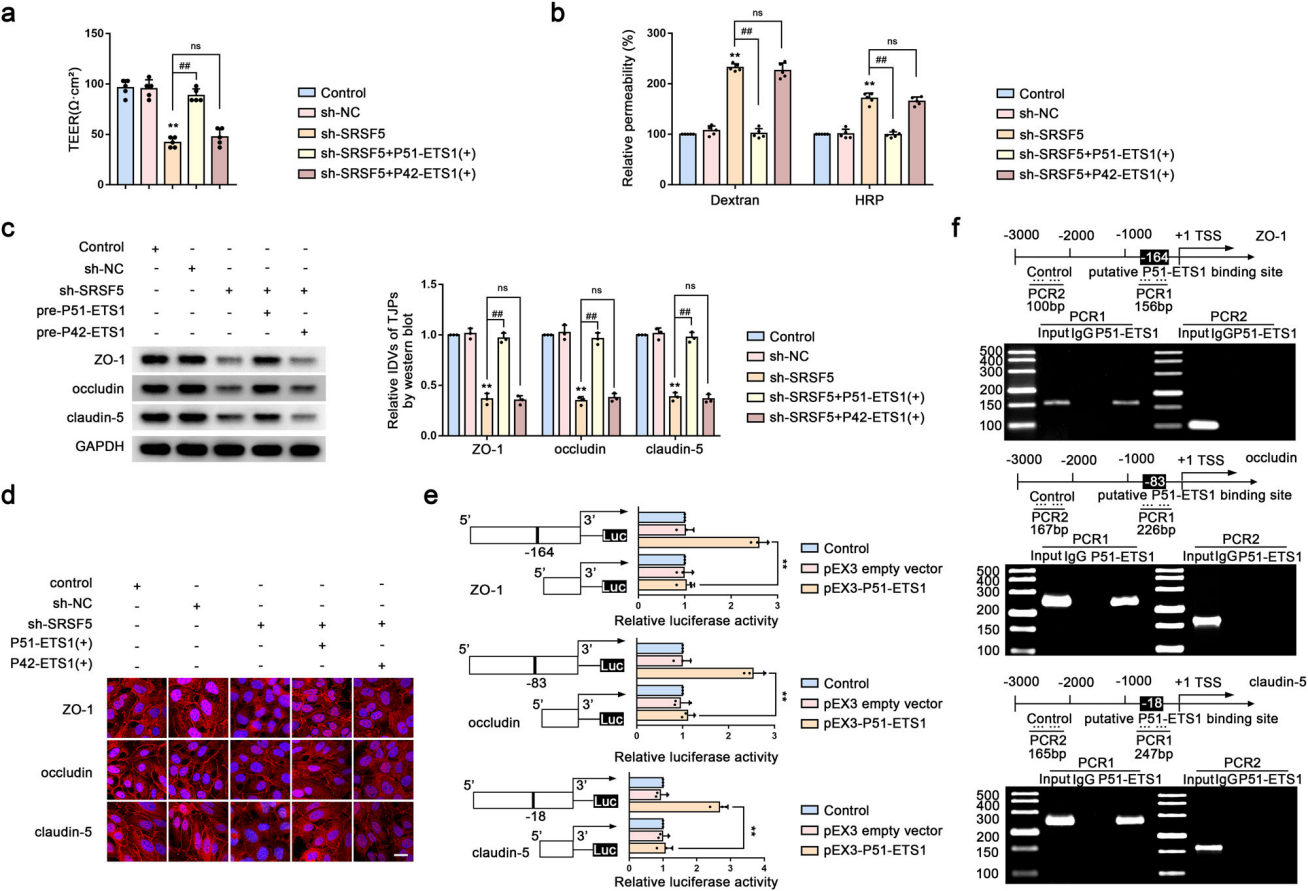
P51-ETS1 Regulates BTB Permeability by Promoting the Transcription of ZO-1, Occludin, and Claudin-5. **a**, **b** The permeability and integrity of the sh-SRSF5, sh-SRSF5 + P51-ETS1( + ) and sh-SRSF5 + P42-ETS1(+) BTB model in vitro were detected by TEER values, FITC-dextran, and HRP flux. Data are represented as mean ± SD (*n* = 5). ^**^P < 0.01 vs. sh-NC group, ^##^*P* < 0.01 vs. sh-SRSF5 group. **c** Effects of sh-SRSF5, sh-SRSF5 + P51-ETS1(+) and sh-SRSF5 + P42-ETS1(+) on the expressions of ZO-1, occludin and claudin-5 were analyzed by western blot assays. Data are represented as mean ± SD (*n* = 3). ^**^P < 0.01 vs. sh-NC group, ^##^*P* < 0.01 vs. sh-SRSF5 group. **d** The distributions of ZO-1, occludin and claudin-5 in the sh-SRSF5, sh-SRSF5 + P51-ETS1( + ) and sh-SRSF5 + P42-ETS1(+) GECs were determined by IF staining. Scale bar represents 50 µm. **e** Dual luciferase reporter assays were performed to determine the binding sites of ETS1 and ZO-1, occludin and claudin-5 in HEK293T cells. **f** ZO-1, occludin and claudin-5 promoter region 3,000 bp upstream of the transcription start sites (TSSs), which were designated as +1. PCR1 represents the binding site of P51-ETS1 to the ZO-1, occludin and claudin-5 promoter region and PCR2 represents the unbound negative control group.

We then established GECs stably expressing sh-SRSF5 + P51-ETS1( + ) and sh-SRSF5 + P42-ETS1( + ), revealing significant increases in the TEER value and TJP levels and significant reductions in the FITC–dextran and HRP-flux signals in the sh-SRSF5 + P51-ETS1 group and no change in the sh-SRSF5 + P42-ETS1( + ) group (Fig. 7a–d). Additionally, dual-luciferase reporter assays confirmed significant increases in the activities of the ZO-1, occludin, and claudin-5 promoters in the pEX3–P51-ETS1 group relative to that in the empty vector group (Fig. 7e). Furthermore, ChIP assays demonstrated that P51-ETS1 directly bound to the promoter regions of ZO-1, occludin, and claudin-5 (Fig. 7f).

### BTB permeability in Dox-treated GBM xenograft mouse models

**Fig. 8:**
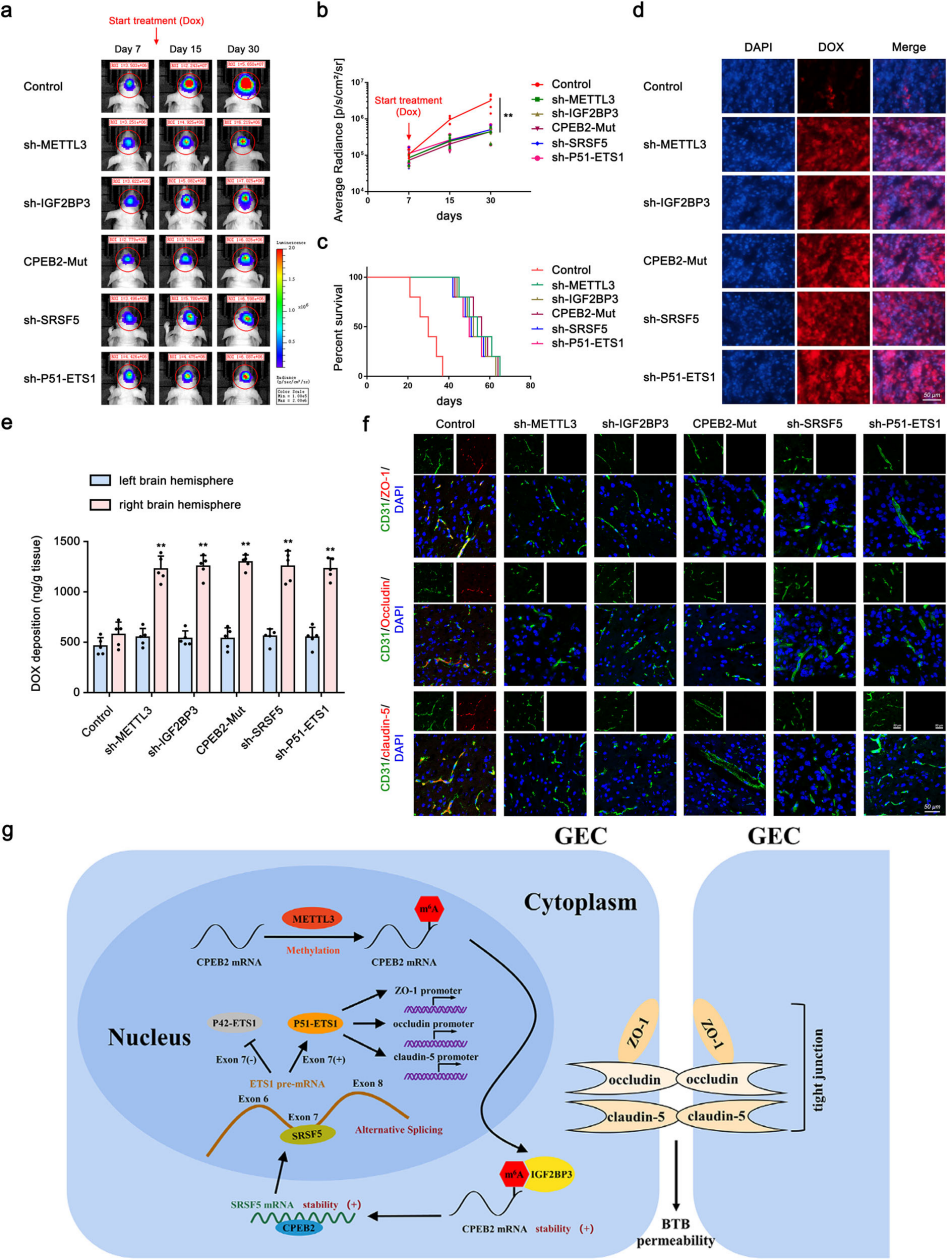
BTB Permeability in Dox-treated GBM Xenograft Mouse Models. **a** BLI of intracranial tumor in mice bearing U251-LUC derived GBM after treatment with Dox on indicated days after GBM implantation. **b** Statistical analyses for the BLI signal intensity of U251 tumors. Data are presented as the mean ± SD (*n* = 5, ^**^P < 0.01 vs. control group). **c** Kaplan-Meier survival curves of mice bearing GBM xenografts with indicated treatments, compared with sh-NC group (*n* = 5 mice/group; two tailed log-rank test, *P* < 0.01). **d** Fluorescence microscope images showing the distribution of DOX in the glioma. DOX distribution is in red and cell nuclei were stained with DAPI (blue). Scale bar, 50 µm. **e** Quantitative analysis of DOX in excised mouse brains. Data are presented as the mean ± SD (*n* = 3, ^**^P < 0.01). **f** Immunofluorescent analyses of CD31 (green) and ZO-1, occludin and claudin-5 (red) in GBM xenografts from mice with indicated treatments. Scale bar, 50 µm. **g** The schematic diagram of the mechanism with which CPEB2 m6A methylation regulates BTB permeability by regulating splicing factor *SRSF5* mRNA stability.

We injected U251-LUC cells into nude mice to establish a model of in situ GBM orthotopically transplanted tumors. After 20 days, we injected sh-METTL3, sh-IGF2BP3, CPEB2-Mut, sh-SRSF5, and/or sh-P51-ETS1 constructs in the tail vein of each mouse, respectively, using the AAV2/9 serotype to enable transfection into mouse cerebral microvessels. After 3 weeks, the mice were treated with Dox once every week. Observation of in situ tumor size using BLI on the treatment day and days 15 and 30 after treatment revealed that Dox treatment after knocking down the target molecules in GECs significantly delayed tumor growth and induced a longer survival time as compared with that in the control group (Fig. 8a–c). Additionally, evaluation of Dox distribution in frozen tumor tissue sections using fluorescence microscopy indicated increased red fluorescence in the sh-METTL3, sh-IGF2BP3, CPEB2-Mut, sh-SRSF5, and sh-P51-ETS1 group, indicating that knockdown of these molecules increased the amount of Dox crossing the BTB into the glioma (Fig. 8d). To quantitatively measure Dox in U251 tumors and normal brain parenchyma, we harvested tumor-bearing and contralateral brain tissues after Dox treatment, followed by HPLC analysis (Fig. 8e). The results showed that Dox deposition in the right brain hemisphere in the sh-METTL3, sh-IGF2BP3, CPEB2-Mut, sh-SRSF5, and sh-P51-ETS1 group was significantly greater than that in the control group. Furthermore, IF staining of ZO-1, occludin, and claudin-5 revealed significant reductions in the TJPs in the sh-METTL3, sh-IGF2BP3, CPEB2-Mut, sh-SRSF5, and sh-P51-ETS1 group (Fig. 8f). Figure 8g provides a diagram of the proposed mechanism in which CPEB2 m6A methylation regulates BTB permeability by regulating *SRSF5* mRNA stability.

## Discussion

The BTB partially or fully inhibits the transport of macromolecular chemotherapeutic drugs to glioma tissue to exert their therapeutic effects. Therefore, regulating the selective permeability of the BTB represents an effective strategy for improving chemotherapy efficacy in glioma. In this study, we identified significant upregulation of METTL3, IGF2BP3, CPEB2, SRSF5, and P51-ETS1 in GECs, whereas their knockdown down significantly reduced the expression of TJPs and increased BTB permeability. METTL3 promotes m6A methylation of *CPEB2* mRNA, whereas IGF2BP3 enhances its stability. CPEB2 subsequently enhances the stability of *SRSF5* mRNA and regulates the SRSF5-mediated alternative splicing of ETS1. The P51-ETS1 splice variant regulates levels of ZO-1, occludin, and claudin-5, thus regulating BTB permeability through the paracellular pathway. We used the TEER value and FITC–dextran and HRP–flux signals as important indicators to evaluate BTB integrity and permeability, with decreases in the TEER value and increases in the FITC–dextran and HRP–flux signals indicating increased BTB permeability^14,15^. In the present study, cytological experiments combined with establishment of a GBM orthotopic xenograft mouse model demonstrated that these proteins regulate BTB permeability.

M6A methyltransferases include METTL3, METTL14, and WT1-associated protein^16–18^. In the present study, we evaluated three NBTs and 10 glioma tissue samples to analyze the differential expression of m6A-related genes and further verified them in the BTB model. We identified METTL3 as highly expressed in GECs, and that its knockdown increased BTB permeability, suggesting that METTL3 participates in regulating BTB permeability. Recent significant progress has been made in elucidating the role of m6A modifications in various stages of the RNA life cycle^19^. For example, m6A reportedly participates in the progression of various cancers^20^, with METTL3 expression identified as upregulated in a variety of tumor tissues and playing both carcinogenic roles and increases in levels of m6A modification. Additionally, high levels of METTL3 have been found in cancers associated with the liver and lungs^21^. To further clarify the role of METTL3, we explored the role of CPEB2, which is targeted by METTL3, using RNA-Seq and MeRIP-seq data from the GEO database, followed by MeRIP-qPCR analysis upon METTL3 knockdown to examine the mechanism of METTL3-mediated m6A modification of *CPEB2* mRNA. CPEB2 binds to the cytoplasmic polyadenylation element in the 3′ UTR to regulate the mRNA translation of target genes and reportedly plays an important role in the development of triple-negative breast cancer^22^. In the present study, we found that CPEB2 was highly expressed in GECs, and that its knockdown increased BTB permeability. Moreover, we found that METTL3 enhanced *CPEB2* mRNA stability. Similar studies in gastric cancer cells showed that METTL3 promotes m6A modification of the mRNA of recombinant protein hepatoma-derived growth factor (HDGF), whereas IGF2BP3 directly recognizes and binds to the m6A site on *HDGF* mRNA, enhancing its stability^23^.

The IGF2BP family includes IGF2BP1, IGF2BP2, and IGF2BP3^24^, with studies reporting IGF2BP3 as highly expressed in a variety of tumors, thereby suggesting it as a potential therapeutic target^25–27^; however, its function in blood vessels has not been reported. In the present study, we found significantly higher IGF2BP3 levels in GECs relative to IGF2BP1 and IGF2BP2 levels. Moreover, after IGF2BP3 knockdown, we identified increases in BTB permeability, suggesting its regulatory role in this process. In eukaryotes, IGF2BPs are recognized as m6A readers, and reports indicate that IGF2BPs regulate the expression of MYC proteins in an m6A-dependent manner in hepatocarcinoma cells^12^. Furthermore, IGF2BP1 promotes serum response factor-dependent transcription in cancer in an m6A-dependent manner^28^, whereas IGF2BP2 targets m6A-containing *differentiation-antagonizing non-protein-coding RNA* (*DANCR*) to enhance its translation, with IGF2BP2 and *DANCR* jointly promoting the occurrence of pancreatic cancer^29^. In the present study, we demonstrated that IGF2BP3 binds and enhances the stability of *CPEB2* mRNA. Interestingly, mutating the *CPEB2* m6A site increased BTB permeability and promoted Dox entry into tumor cells.

Alternative splicing is among the most common gene-regulation mechanisms and plays an important role in the complex regulation of protein functions. Splicing dysregulations are closely related to the occurrence and development of many tumors in humans^30^. Additionally, alternative splicing of pre-mRNA is ubiquitous in mammalian cells, with ~95% of human mRNA being formed by alternative splicing^31–33^, which helps regulate gene expression and expand proteome diversity. SR proteins are widely regarded as active splicing regulators and promote exon inclusion^34^. Among them, SRSF1 promotes the inclusion of CD33 exons in Alzheimer’s disease to enhance the transcription and expression of full-length CD33 and regulates their specific interaction with *CD33* pre-mRNA, thus altering the protein levels on the cell surface^35^. Moreover, SRSF6 reportedly increases the inclusion of *OGDHL* exon 3, thereby affecting pancreatic cancer cell metastasis^36^. SRSF5 contains two N-terminal RNA-recognition motifs and an arginine/serine-enrichment domain and plays an important regulatory role in RNA splicing and translation^37^. Abnormal expression of SRSF5 was reported in breast, renal, and lung cancers^38^, and studies identified SRSF5 involvement in the alternative splicing of *HSD17B2* mRNA in prostate cancer and its regulation of tumor growth via alternative splicing of *CCAR1* pre-mRNA in lung cancer^39–41^. In the present study, we found significant decreases in SRSF5 levels following CPEB2 knockdown, suggesting that *SRSF5* mRNA might be a CPEB2 target. Additionally, we identified elevated SRSF5 levels in GECs and noted that BTB permeability increased upon SRSF5 knockdown, suggesting the involvement of SRSF5 in regulating BTB permeability. We further confirmed the binding affinity of CPEB2 with *SRSF5* mRNA, and that CPEB2 knockdown significantly reduced SRSF5 levels and shortened the *SRSF5* mRNA half-life (although levels of nascent *SRSF5* mRNA remained unchanged), suggesting that CPEB2 might regulate BTB permeability by binding to *SRSF5* mRNA and increasing its stability.

We detected possible SRSF5 targets through correlation analysis using TCGA data and subsequently showed that ETS1 level was significantly decreased following SRSF5 knockdown, suggesting ETS1 as an SRSF5 target. ETS1 is involved in regulating tumor cell proliferation, development, apoptosis, metastasis, invasion, and angiogenesis^42^, and its ETS domain (transcription-activation domain) and helical DNA-binding domain are involved in regulating the maturation of vascular ECs and endothelial barrier function^43^. ETS1 is highly expressed in a variety of tumor tissues, and inhibiting ETS1 can block tumor proliferation, migration, and invasion in vivo^44,45^. Moreover, expression of ETS-family transcription factors is essential for EC differentiation, with ETS1 and ETS2 affecting tumor angiogenesis and metastasis in the tumor microenvironment, especially in ECs^46,47^. Among the splice variants of mouse Ets1, P42-ETS1 and P51-ETS1 were identified as transcription factors with different targets and activities^48^. For example, MDA-MB-231 breast cancer cells express P51-ETS1 but not P42-ETS1, with only 10% of primary breast cancer cells simultaneously expressing P51-ETS1 and P42-ETS1^49^. In the present study, we found that in contrast to P42-ETS1, P51-ETS1 was highly expressed in GECs, and that its knockdown increased BTB permeability and overexpression reversed this effect. Conversely, P42-ETS1 overexpression had no effect on BTB permeability, suggesting that P51-ETS1 participates in regulating BTB permeability. We further verified that P51-ETS1 binds to the promoter regions of *ZO-1*, *occludin*, and *claudin-5* to increase their transcription levels. Furthermore, we identified that SRSF5 promotes the inclusion of *ETS1* exon 7 and regulates BTB permeability in GECs, which further elucidated the function of SR proteins in alternative splicing.

Dox is an anthracycline antitumor antibiotic used in the clinical treatment of malignant tumors^50^. However, Dox has difficulties entering the brain parenchyma and reaching an effective therapeutic concentration for the treatment of glioma due to the BBB^51^. A previous study showed that the combined application of KHDRBS3 can promote the transmembrane transport of Dox and induce apoptosis of glioma cells^52^. To further evaluate the regulatory effects of these factors on BTB permeability, we used GBM orthotopic xenograft mice to demonstrate that mutation of m6A sites in *CPEB2* mRNA and METTL3, SRSF5, and P51-ETS1 knockdown in GECs combined with Dox administration significantly increased BTB permeability, significantly reduced the size of GBM-transplanted tumors, prolonged survival, and increased the amount of Dox crossing the BTB into tumors. Low passive paracellular permeability and high expression levels of active efflux drug transporters in BBB together limit the exposure of many anticancer drugs to the brain^53,54^. Increased passive permeability does not always equate to increased drug accumulation, as P-glycoprotein efflux transporters are still active in tumors despite disrupted vasculature^55,56^. Thus, the link between METTL3 or IGF2BP3 and efflux transporters deserves further investigation.

In summary, we found that upregulated levels of METTL3 and IGF2BP3 in GECs increase the stability and expression of *CPEB2* mRNA via m6A methylation. This enables CPEB2-mediated increases in the stability of *SRSF5* mRNA to promote *ETS1* exon-7 inclusion and formation of the P51-ETS1 spliceosome, which stimulates transcription of ZO-1, occludin, and claudin-5 to regulate BTB permeability. Furthermore, in vivo knockdown of these proteins in GBM xenograft mice enhanced the entry of Dox through the BTB and promoted the apoptosis of glioma cells. These findings provide a theoretical and experimental basis for the epigenetic regulation of the BTB, as well as strategies for the comprehensive treatment of gliomas.

## Methods

### The Cancer Genome Atlas (TCGA) and Genotype–Tissue Expression Project (GTEx) database analysis

Data from glioma patients concerning gene expression, correlational, and prognosis were obtained from TCGA (https://portal.gdc.cancer.gov/). Normal brain tissue (NBT) data were obtained from the GTEx database (https://www.gtexportal.org/home/). All analyses were performed in R (https://www.r-project.org/).

### Laser capture microdissection (LCM)

All human glioma specimens and NBT were obtained from the Department of Neurosurgery of Shengjing Hospital, China Medical University. All participants signed and provided informed consent, and this study was approved by the Institutional Review Board of Shengjing Hospital of China Medical University. Surgical human brain specimens of tissues, low-grade glioma (LGG), and high-grade glioma (HGG) were frozen and sectioned at 10-μm thickness using a microtome/cryostat (Microm International, Walldorf, Germany). Subsequently, LCM was performed, as previously described^57^. Sections of vessels in glioma tissue (or NBT) were stained using the *Ulex europaeus* agglutinin I (UEA-I) fluorescent dye-tagged lectin (Vector Laboratories, Burlington, ON, Canada) according to manufacturer instructions. LCM was then conducted using the ArcturusXT microdissection instrument. Captured microvessels were transferred into CapSure LCM caps (Applied Biosystems, Foster City, CA, USA) and further processed for RNA isolation.

### Cell lines and cell culture

The hCMEC/D3 cells (ECs) immortalized human brain endothelial cell line was provided by Dr. Couraud (Cochin Institute, Paris, France) and cultured on culture inserts (0.4-µm pore size; Corning, Lowell, MA, USA) coated with 150 μg/mL Cultrex rat collagen I (R&D Systems, Minneapolis, MN, USA). Cells were cultured in endothelial base medium (EBM-2; Lonza, Walkersville, MD, USA) containing 5% fetal bovine serum (FBS) “Gold” (PAA Laboratories, Pasching, Austria), 1% penicillin–streptomycin (Life Technologies, Paisley, UK), 1.4 mol/L hydrocortisone (Sigma-Aldrich, St Louis, MO, USA), 1% lipid concentrate (Life Technologies), 5 g/mL ascorbic acid (Sigma-Aldrich, St Louis, MO, USA), 10 mmol/L HEPES (PAA Laboratories GmbH), and 1 ng/mL human basic fibroblast growth factor (Sigma-Aldrich). ECs were maintained for no more than 30 passages. The U251 human glioblastoma and HEK293T cell lines were purchased from the Cell Resource Center of Shanghai Institute of Biological Sciences (Shanghai, China) and stored in Dulbecco’s modified Eagle medium containing 10% FBS, 100 U/mL penicillin, and 100 μg/mL streptomycin (Life Technologies). Normal human astrocytes (NHAs) were purchased from ScienCell Research Laboratories (Carlsbad, CA, USA) and cultured in the astrocyte culture medium Roswell Park Memorial Institute-1640 (ScienCell Research Laboratories). The U251 cell line was previously tested for mycoplasma contamination and authenticated by short tandem-repeat DNA profiling. All cells were cultured in a humidified incubator at 37 °C and 5% CO_2_.

### Establishment of an in vitro BTB and Blood–Brain Barrier (BBB) model

The in vitro BTB model was established by co-cultivation of ECs and U251 cells, as previously described^58^. U251 cells were seeded in 6-well plates at a density of 2 × 10^4^ cells/well and cultured for 2 days. ECs were seeded at a density of 2 × 10^5^ cells/well on an insert coated with Cultrex rat collagen I (R&D Systems), which was then placed in the 6-well plates. After co-culturing for 4 days to reach confluence, glioma microvascular ECs (GECs) were obtained. Both ECs and U251 cells were cultured with the prepared EBM-2 medium, which was changed every 2 days. The in vitro BBB model used the same method of co-culturing ECs with NHAs to obtain microvascular ECs (AECs).

### Real-time PCR (qRT-PCR) assays

Total RNA was extracted using TRIzol reagent (Life Technologies), and a SYBR PrimeScript primary RT-PCR kit (Takara Bio, Beijing, China) was used to evaluate RNA-expression levels using the 7500 Fast RT-PCR system (Applied Biosystems), with GAPDH used as an endogenous control. Relative expression was calculated using the 2^−ΔΔCt^ method. The primers are provided in Supplementary Table 1.

### Western blot assays

Western blot assays were performed as previously described^59^. For details of the experiment, please refer to the Supplementary Information. The primary antibodies used for western blotting were as follows: METTL3 (15073-1-AP; 1:500 dilution; Proteintech), IGF2BP3 (14642-1-AP; 1:500 dilution; Proteintech), CPEB2 (ab51069; 1:500 dilution; Abcam), SRSF5 (ab67175; 1:500 dilution; Abcam), ETS1 (sc-55581; 1:500 dilution; Santa Cruz Biotechnology), ZO-1 (61-7300; 1:500 dilution; Thermo Fisher Scientific), occludin (71-1500; 1:500 dilution; Thermo Fisher Scientific), claudin-5 (35-2500; 1:500 dilution; Thermo Fisher Scientific), GAPDH (60004-1-lg; 1:10000 dilution; Proteintech). The antibody used are provided in Supplementary Table 3.

### Cell Transfection

Cell transfections were performed as previously described^14^. ECs were seeded in 24-well plates and transfected using Opti-MEM I and Lipofectamine LTX reagents (Life Technologies) under fusion conditions of ~80% according to manufacturer instructions. Stable cell lines were selected using geneticin (G418) or puromycin. After 4 weeks of application, G418-resistant (or puromycin-resistant) clones were obtained. Plasmids and corresponding empty vectors were constructed using GenePharma (Shanghai, China). Protein knockdowns were confirmed using western blot (Fig. S2). The CPEB2 m6A site mutation was introduced using the QuikChange site-directed mutagenesis kit (Agilent Technologies) according to manufacturer instructions. The target sequences and plasmid vectors are shown in Supplementary Table 2.

### Transendothelial electrical resistance (TEER) assays

The TEER value was measured using a Millicell-ERS instrument (Millipore, Billerica, MA, USA) after establishing the in vitro BTB model. The media in the upper and lower chambers of the Transwell were replaced with fresh media, and the TEER value was measured after 30 min at 25 °C. We obtained the final resistance (Ωcm^2^) by subtracting the background resistance from the measured blocking resistance and then multiplying it by the effective surface area of the filter.

### Horseradish peroxidase (HRP) flux assays

After establishing the in vitro BTB model, 0.5 µmol/L HRP (Sigma-Aldrich) was added to the upper chamber of the Transwell, and incubated for 1 h at 37 °C. Then 200 µL TMB color developing solution and 5 µL small chamber culture medium were added to a 96-well plate and incubated at ambient temperature for 30 min. The OD value of each 96-well sample was measured using a microplate reader to calculate the HRP content of the lower chamber.

### Fluorescein isothiocyanate (FITC)–dextran permeability assays

FITC–dextran (4 kDa; Sigma-Aldrich) was added to the upper chamber of the Transwell insert (2 mg/mL) to assess in vitro BTB permeability. The medium was collected from the lower chamber of the Transwell insert after 1 h of incubation, and a multi-function microplate reader was used to measure the FITC–dextran content.

### Immunofluorescence (IF) assays

Cell slides were fixed with paraformaldehyde for 30 min and washed three times with phosphate-buffered saline with Tween 20 (PBST). After penetrating the membrane with Triton X-100 for 10 min, the slides were washed again with PBST. After blocking with 5% bovine serum albumin for 2 h, the slides were incubated with the corresponding antibodies for ZO-1, occludin, and claudin-5 overnight. After reheating at 25 °C for 30 min, the primary antibody was washed with PBST, and the corresponding secondary antibody was applied at 25 °C for 2 h. Three washes with PBST for 10 min were then performed. The nuclei were stained with DAPI for 5 min. After the staining was completed, the PBST wash was performed three times, and the slides were sealed with 50% glycerol. Slides were then observed and photographed under a confocal microscope.

### Methylated RNA immunoprecipitation (MeRIP)-qPCR assays

Cells were incubated with an anti-m6A antibody (ab208577; Abcam) at 4 °C for 1 h and then mixed with pre-washed Pierce protein A/G magnetic beads (88,803; Thermo Fisher Scientific) in immunoprecipitation buffer at 4 °C overnight. The m6A antibody was digested with proteinase K digestion buffer, and methylated RNA was purified for qRT-PCR analysis.

### M6A dot blot assays

PolyA(+) RNA was first denatured by heating at 65 °C for 5 min and then transferred to a cellulose nitrate membrane (Amersham, GE Healthcare, USA) using a Bio-dot device (Bio-Rad, USA). The membranes were then UV-crosslinked, sealed, and incubated overnight at 4 °C with m6A antibody (1:1000, Abcam) and then incubated with HRP-conjugated goat anti-mouse IgG (1:300, Proteintech, USA). The membranes were then visualized by enhanced chemiluminescence (Bio-Rad). The membrane was stained with 0.02% Methylene Blue (MB) in 0.3 M sodium acetate (pH 5.2) to ensure consistency across groups.

### RNA immunoprecipitation (RIP) and RNA pull-down assays

For details on these experiments, please refer to the Supplementary Methods.

### Nascent RNA capture

Nascent RNA was detected using the Click-iT nascent RNA capture kit (Thermo Fisher Scientific). 5-Ethynyl uridine (EU) was incorporated into nascent RNAs, followed by RNA capture using streptavidin magnetic beads and qRT-PCR analysis.

### RNA stability measurement

Cells were cultured in medium containing 5 µg/mL actinomycin D (Act D, NobleRyder, China). Subsequently, total RNA was extracted at different time points and detected via qRT-PCR. Compared with the zero time point, the half-life of RNA was determined according to the level of RNA reduced to 50% at a set time point.

### Minigene assays

The *ETS proto-oncogene 1 (ETS1)* exon7 minigene was constructed by inserting the exons and flanking intron region of ETS1 into the pGint Vector. The ETS1 exon7 and its flanking intron region were amplified from genomic DNA using the ETS1 minigene-F and minigene-R primers. The specific primers for complete EGFP RNA were used to determine the splicing efficiency by qRT-PCR. Primers are shown in Supplementary Table 1.

### Chromatin immunoprecipitation (ChIP) assays

ChIP assays were performed using the Simple ChIP enzymatic chromatin IP kit (Cell Signaling Technology, Danvers, MA, USA) according to manufacturer instructions. For details on this experiment, please refer to the Supplementary Methods. Primers used for ChIP PCR are shown in Supplementary Table 4.

### Establishment of the orthotopic brain glioblastoma xenograft model

For the animal studies, a protocol detailing experimental procedures following the China Medical University guidelines was submitted to and approved by Ethics Committee of China Medical University. Female Balb/c nude mice (8-weeks old) were purchased from HFK Bioscience (Beijing, China). U251-LUC cells stably expressing luciferase constructs (1 × 10^6^ cells/mouse) were injected into the caudate nucleus of the right brain hemisphere of nude mice. Recombinant AAV2/9 was used to repress gene expression in mice cerebral microvascular ECs. Short-hairpin (sh)RNA sequences were ligated into pAKD-CMV-bGlobin-eGFP-H1-shRNA (Obio Technology, Shanghai, China). The sequences are shown in Supplementary Table 2. For details on this experiment, please refer to the Supplementary Methods.

### Bioluminescence in vivo imaging (BLI)

Mice were intraperitoneally injected with 200 μL of 150 mg/kg D-fluorescein (Promega, Madison, WI, USA) and then anesthetized with isoflurane after 5 min. Animals were imaged using the IVIS Lumina II imaging system (Xenogen, Alameda, CA, USA).

### Dox uptake by tumors

After 2 h of Dox treatment on day 30, brain tissue was collected after anesthesia and perfusion and divided into left (control) and right (tumor-tissue-containing) brain hemispheres. Brain samples were then homogenized and soaked in acidified ethanol (50% ethanol in 0.3 N hydrochloric acid) for 24 h at 4 °C to completely extract Dox. A freezing centrifuge was then used to centrifuge samples at 14,000 rpm for 10 min. Dox concentration in the clear supernatant was analyzed by high-performance liquid chromatography (HPLC) and expressed as Dox per gram of tissue.

### Histological analysis

At the end of treatment, mice were sacrificed, and the tumors were excised for histologic analysis. Brain tissue was collected, prepared, and sectioned according to standard procedures. The tissue was embedded in OCT compound and cut into 8-µm-thick sections for IF staining. Fluorescence microscopy was used to qualitatively assess the permeability to Dox.

### Statistics and reproducibility

Statistical analysis was performed using Student’s *t* test or one-way ANOVA in GraphPad Prism7, and data are presented as the mean ± standard deviation (SD). *P* < 0.05 was considered significant. The number of samples per independent experiment are described in the legends.

### Reporting summary

Further information on research design is available in the Nature Research Reporting Summary linked to this article.

## Supplementary information

Supplementary Information

Description of Additional Supplementary Files

Supplementary Data 1

Reporting Summary

## Data Availability

All data that support the findings of this study are available from the corresponding author upon reasonable request. Full-length uncropped original western blots used in the manuscript are shown in Supplementary Information. The numerical data that make up the all graphs in the paper are shown in Supplementary Data 1.
