# Supplementary material for: CPEB2 m6A methylation regulates blood–tumor barrier permeability by regulating splicing factor SRSF5 stability
Source: Commun Biol. 2022 Sep 5;5:908. doi: 10.1038/s42003-022-03878-9 (PMC9445078; doi:10.1038/s42003-022-03878-9)
Supplement: Supplementary file 1 — Supplementary Information [file 42003_2022_3878_MOESM1_ESM.pdf]

Supplementary Information

CPEB2 m6A Methylation Regulates Blood–Tumor Barrier Permeability by Regulating Splicing Factor SRSF5 Stability

Authors: Mengyang Zhang[1,2,3], Chunqing Yang[4,5,6,] Xuelei Ruan[1,2,3], Xiaobai Liu[4,5,6], Di Wang [4,5,6], Libo Liu[1,2,3], Lianqi Shao[1,2,3], Ping Wang[1,2,3], Weiwei Dong[4,5,6], Yixue Xue[1,2,3*]

Affiliations:

[1]Department of Neurobiology, School of Life Sciences, China Medical University, Shenyang 110122, People's Republic of China;

[2]Key Laboratory of Cell Biology, Ministry of Public Health of China, China Medical University, Shenyang 110122, People's Republic of China;

[3]Key Laboratory of Medical Cell Biology, Ministry of Education of China, China Medical University, Shenyang 110122, People's Republic of China;

[4]Department of Neurosurgery, Shengjing Hospital of China Medical University, Shenyang 110004, People's Republic of China;

[5]Liaoning Research Center for Translational Medicine in Nervous System Disease, Shenyang 110004, People's Republic of China;

[6]Key Laboratory of Neuro-oncology in Liaoning Province, Shenyang 110004, People's Republic of China

Correspondence: Yixue Xue, Department of Neurobiology, College of Life Sciences,

China Medical University, Shenyang 110122, People's Republic of China.

E-mail: xueyixue88cmu@163.com

This file includes:

Supplementary Figures 1-2

Supplementary Methods

Supplementary Tables 1-4

Uncropped original western blots

Supplementary Figures

Figure S1. METTL3 was highly expressed in glioma tissues and GECs

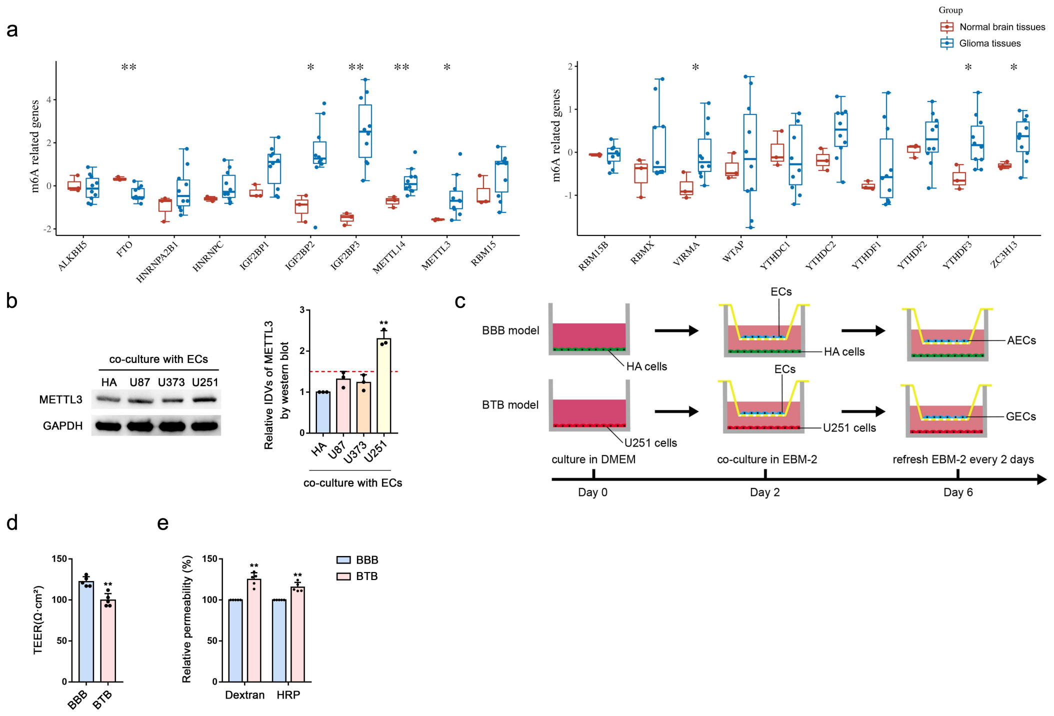

Figure S1 (a) The expression distribution of m6A mRNA in case and control groups, where the horizontal axis represents different mRNA, the vertical axis represents the mRNA expression distribution, where different colors represent different groups, and the upper left corner represents the significance p-value test method. Asterisks represent levels of significance $^{*}P < 0.05$, $^{**}P < 0.01$, $^{***}P < 0.001$. **(b)** Relative protein levels of METTL3 in AECs (ECs co-cultured with HA) and GECs (ECs co-cultured with U87, U373 and U251 respectively) were determined by western blot assays. Data represented as mean ± SD (n = 3). $^{**}P < 0.01$ vs. AECs group. **(c)** A schematic showing in vitro BBB and BTB model. **(d, e)** The permeability and integrity of BBB and BTB model *in vitro* were detected by TEER values, 4-kDa FITC-dextran leakage and HRP flux. Data represented as mean ± SD (n = 5). $^{**}P <$ 0.01 vs. BBB group.

Figure S2. Confirmation of protein knockdowns

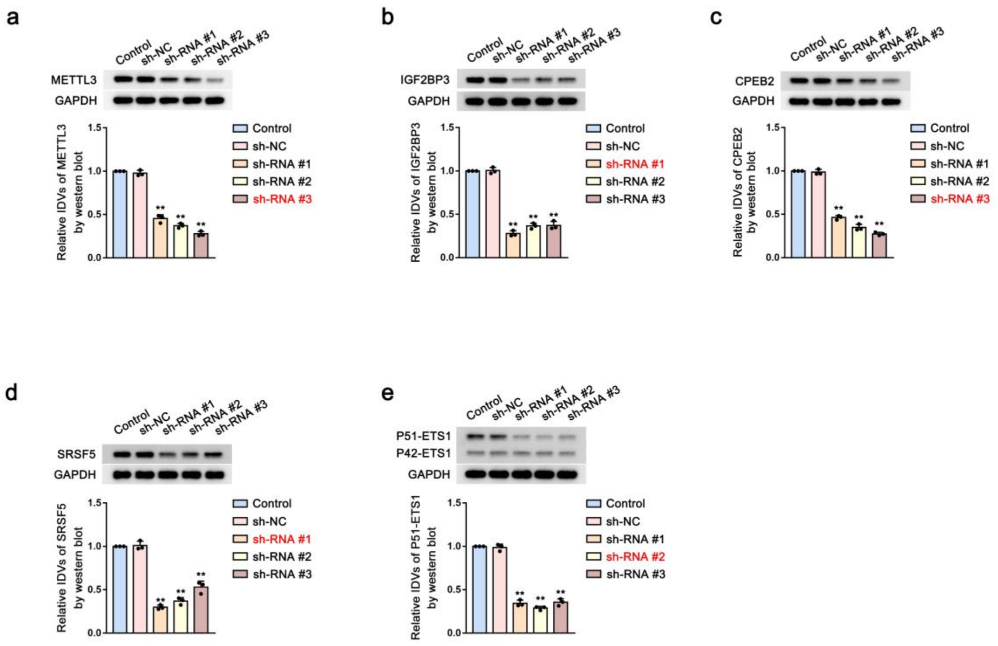

Figure S2 (a-e) Relative protein levels were determined by western blot assays. Data represented as mean ± SD (n = 3). $^{**}P < 0.01$ vs. sh-NC group. The selected sh-RNA plasmids were shown in red.

Supplementary Methods

Western Blot Assays

The total protein of the cells was extracted with RIPA buffer (Beyotime Institute of Biotechnology, Jiangsu Province) supplemented with protease inhibitors (10 mg/mL aprotinin, 10 mg/mL PMSF and and 50 mM sodium orthovanadate). The protein concentration was then determined using the BCA protein assay kit (Jiangsu Beiyang Institute of Biotechnology, China). The same amount of protein was loaded for SDS-PAGE electrophoresis and then transferred to Millipore (Shanghai, China) and sealed in Tris buffer/Tween 20 (TBST) containing 5% skimmed milk powder for 2 h at room temperature. Incubate primary antibody overnight at 4°C. The membrane was washed three times with TBST and then incubated at room temperature with conjugated HRP secondary antibodies for 2 hours. ECL (enhanced chemiluminescence kit, Santa Cruz Biotechnology, Dallas, TX) detection system (Thermo Scientific, Beijing, China) was used for detection. Scan with Chemi Imager 5500 V2.03. GAPDH was used as internal reference to determine the expression level of the target protein. Antibody used are provided in Supplementary Table 3.

RNA Immunoprecipitation (RIP) Assay

Whole cell lysates from different groups were collected and incubated with magnetic bead RIP buffer containing antibody overnight. The negative control group was incubated with normal mouse IgG (Millipore). Samples were incubated with proteinase K buffer to isolate immunoprecipitated RNA. The RNA concentration was measured using a spectrophotometer (NanoDrop, Thermo Scientific), and the RNA quality was evaluated using a bioanalyzer

(Agilent, Santa Clara, CA, USA). Then purified and reverse transcribed the RNA, detect RNA enrichment by qRT-PCR.

RNA Pull-down Assays

RNA pull-down assays were performed using Pierce Magnetic Magnetic protein pull-down kit (Thermo Fisher) according to the manufacturer's instructions. The retrieved protein was analyzed by western blot with GAPDH as the control.

ChIP Assays

ChIP assays were performed using the Simple Chip Enzymatic Chromatin IP kit (Cell Signaling Technology, Danvers, MA, USA) according to the manufacturer's instructions. GECs were crosslinked with EBM-2 containing 1% formaldehyde for 10 minutes, and incubated with glycine at room temperature for 5 minutes to stop the reaction crosslinking. Add PMSF containing lysate to the cells in the dish to fully lyse. The chromatin in the lysed cells is digested with nuclease. The immunoprecipitation was incubated with 2 μg P51-ETS1 antibody (Santa, USA). Protein G agarose beads were subjected to a cross-linking reaction with their DNA while 2% of the lysate was used as a positive control Input. Add 5 mol/L NaCl and proteinase K to the DNA cross-linking solution to purify the DNA. The purified DNA was used for PCR amplification to verify the binding of P51-ETS1 to the promoter regions of ZO-1, occludin, and claudin-5. Primers used for ChIP PCR are shown in Supplementary Table 4.

Orthotopic Brain Glioblastoma Xenograft

Female 8-week-old nude mice were purchased from HFK Bioscience (Beijing, China). U251-LUC cells stably expressing the indicated luciferase constructs (10^6 cells/mouse) were injected into the caudate nucleus of the right brain hemisphere of nude mice. Nude mice were anesthetized by intraperitoneal injection of 0.8% sodium pentobarbital (20 $mL \cdot kg^{-1}$), and their heads were fixed on the brain stereotaxic device in the prone position. After disinfecting the skin on the top of the head with 75% ethanol, a vertical incision was made before the eye split at the skull midline to expose the bregma. The inoculation site was 2 mm lateral to the sagittal suture and 1 mm anterior to the bregma. A 1-mm-diameter animal skull drill was used to carefully drill through the skull and a microinjector to inoculate 5 μL tumor cell suspension entering the white matter area vertically through the hole. The needle was inserted to a depth of 4 mm from the needle tip to the skull surface. Before injection, the needle was slightly retracted by approximately 1 mm, and the tumor cell suspension was slowly injected at a 0.5-$\mu L \cdot min^{-1}$ injection rate; the needle was kept inside the skull for 3 minutes. Then, it was slowly pulled out, the bone hole sealed with bone wax, the surgical field rinsed with saline, and the scalp sutured and disinfected with iodine. After the operation, the nude mice were placed on a constant temperature plate at 37°C for heat preservation and then returned to the cage after awakening. No anti-infectious treatment was required after the operation. The life status of mice was observed every day, including mental diet and physical activity, among other parameters. Recombinant AAV2/9 was used to repress gene expression in mice cerebral microvascular ECs. Short-hairpin (sh)RNA sequences were ligated into pAKD-CMV-bGlobin-eGFP-H1-shRNA (Obio Technology, Shanghai, China). The sequences

are shown in Supplementary Table 2.

Supplementary Table 1. Primers used for qRT-PCR

Gene	Sequence (5'->3') or Assay ID
METTL3	F: CTTCAGCAGTTCCTGAATTAGC
	R: ATGTTAAGGCCAGATCAGAGAG
METTL14	F: ACCAAAATCGCCTCCTCCCAAATC
	R: AGCCACCTCTTTCTCCTCGGAAG
IGF2BP3	F: GAGGCGCTTTCAGGTAAAATAG
	R: AATGAGGCGGGATATTTCGTAT
KLF6	F:CTGCAGGAAAGTTTACACCAAA
	R:ACTCATCACTTCTTGCAAAACG
CPEB2	F: GTTCTGCGGCGAGGCGTATG
	R: CAACGGTGGTGGCGACAGTG
TMED9	F:GAGACCATGGTCATAGGAAACT
	R:TATGGGAAGTGAAAGTGAACCT
SRSF1	F: GTTCTACAAATACGGCGCTATC
	R: GACGGTACCCATCGTAATCATA
SRSF2	F: ACAACCTGACCTACCGCACCTC
	R: TGAAAGCGAACGAAGGCGAAGC
SRSF5	F: CAGACCTCGAAATGATAGACGA
	R: CCAGCTGACTCTTGAGGATAAA
SRSF4	F: CTCGCACAGAGTACAGACTTAT
	R: CTTGTGAGCATCTGCATAAGTC
SRSF6	F: CTAAGATGACTGCCTTTCCTGA
	R: TAAGGTCAGCCAAAGGGTCATA
SRSF7	F: AGAACTGTATGGATTGCGAGAA
	R: CACAAATCACCTTTCCATCCAG
MYBL2	F: TCAGAAGTACTCCATGGACAAC
	R: GTCCTCGATGATGAGTTCGAT
AR	F: CTACATCAAGGAACTCGATCGT
	R: CATGTGTGACTTGATTAGCAGG
ETS1	F: TTGAAAGCATAGAGAGCTACGA
	R: CTCTGAGTCGAAGCTGTCATAG
BRD3	F: ATGCAGAATGTGGTGGTGAAG
	R: ATAATCCGGCAGGTTCAATTTG
THAP11	F: ATCGATCTCACAGTGCAAGTG
	R: TGACGACAAGGAGTACGAATG
GAPDH	F: GGTGAAGGTCGGAGTCAACG
	R: CCATGTAGTTGAGGTCAATGAAG
EGFP	F: CCTCGTGACCACCCTGACCTAC
	R: TTGCCGTCGTCCTTGAAGAAGATG

Supplementary Table 2. shRNA and plasmid sequences

Gene	Sequence
METTL3	5'-3':GCAAGAATTCTGTGACTATGG
IGF2BP3	5'-3':GCAAAGGATTCGGAAACTTCA
CPEB2	5'-3':GTGTTCAGAACAGACAACAAT
SRSF5	5'-3':GGATATGGACGGATAAGAGAT
P51-ETS1	5'-3':GGACCGTGCTGACCTCAATAA
P42-ETS1	5'-3':GGACCAGTCGTGGCAGTGGAC

Gene	plasmid sequences
CPEB2-Wt	CATCTTTATCAGCAGCCAAAACACTACAAGCCTCTTGTTTT
CPEB2-Mut	CATCTTTATCAGCAGCCAAACCACTACAAGCCTCTTGTTTT

Supplementary Table 3. Antibodies used for western blotting (WB), RNA-binding protein immunoprecipitation (RIP)

Protein	Applications	Antibody	Origin	Dilution	Molecular weight
METTL3	WB	15073-1-AP, Proteintech	Rabbit	1:500	64kD
IGF2BP1	WB	22803-1-AP, Proteintech	Rabbit	1:500	65kD
IGF2BP2	WB	11601-1-AP, Proteintech	Rabbit	1:500	66kD
IGF2BP3	WB,IP	14642-1-AP, Proteintech	Rabbit	1:500;1μg per IP	69kD
CPEB2	WB,IP,IF	ab51069, Abcam	Rabbit	1:500;1μg per IP;1:50	66kD
SRSF5	WB,IP	ab67175, Abcam	Mouse	1:500;1μg per IP	65kD
P51-ETS1	WB,IP	sc-55581,Santa Cruz	Mouse	1:500;1μg per IP	51kD
P42-ETS1	WB	sc-55581,Santa Cruz	Mouse	1:500	42kD
GAPDH	WB	60004-1-lg, Proteintech	Mouse	1:10000	36kD
IgG	RIP	ab18413, Abcam	Mouse	1:10	150kD
ZO-1	WB,IF	61-7300, Thermo Fisher	Rabbit	1:500;1:50	220kD
Occludin	WB,IF	71-1500, Thermo Fisher	Rabbit	1:500;1:50	59kD
Claudin-5	WB,IF	35-2500, Thermo Fisher	Mouse	1:500;1:50	22kD
Goat anti-mouse	WB	SA00001-1, Proteintech		1:10000	
Goat anti-rabbit	WB	SA00001-2, Proteintech		1:10000	

Supplementary Table 4. Primers used for ChIP

Gene	Sequence (5'->3') or Assay ID
ZO-1 PCR1	F: CTACAGGTGCACACCACCAC
	R: GTGGCTCACGCCTGTAATC
ZO-1 PCR2	F: TTGTGAGGCTGTTGGCTTACT
	R: TCTTGGTGAGTACAGTGAGCTTT
occludin PCR1	F: GTGGAAGTGTGCGCATGTAG
	R: CCTCGACCCTCAAGTTTGTC
occludin PCR2	F: TCTGAGTCACGGGGATTTTC
	R: GGATGTTAAATGATGCCATGC
claudin-5 PCR1	F: GCACTCAGGAAAGGCAGAAG
	R:GATGTGAGCAGCATCCAGAG
claudin-5 PCR2	F: CTACTTGGGAGGCTGAGGTG
	R: TTCTCTGGGTTGCCTTTTTG

Full length uncropped original western blots used in the manuscript

Figure 1f

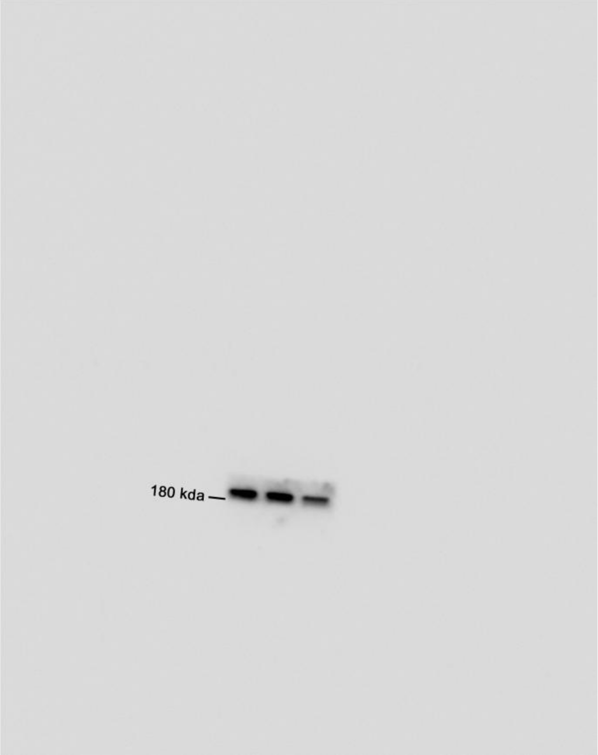

ZO-1

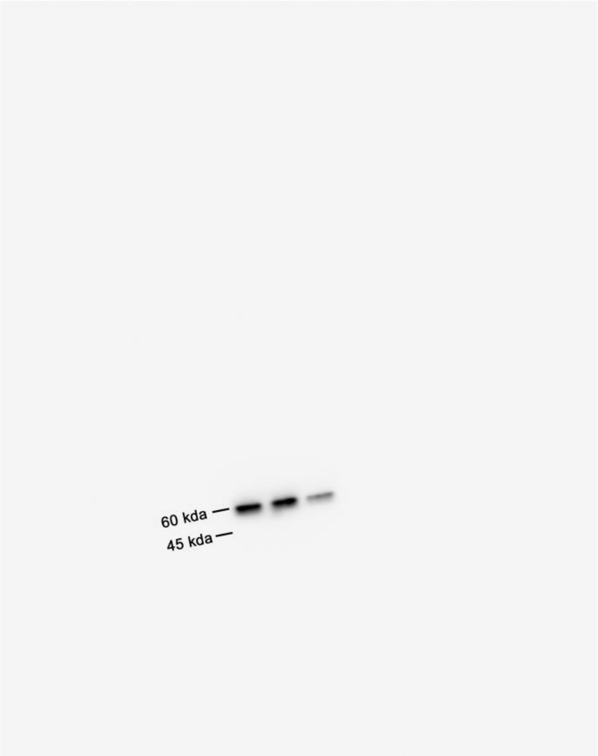

occludin

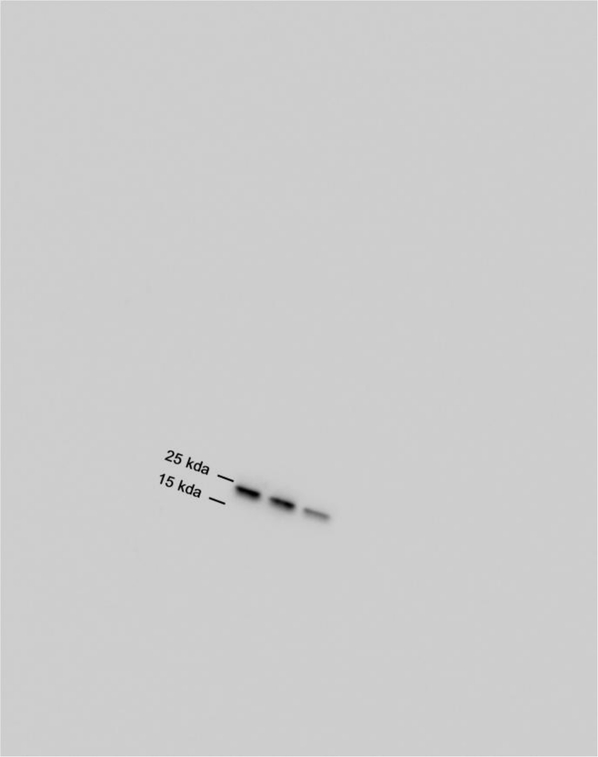

claudin-5

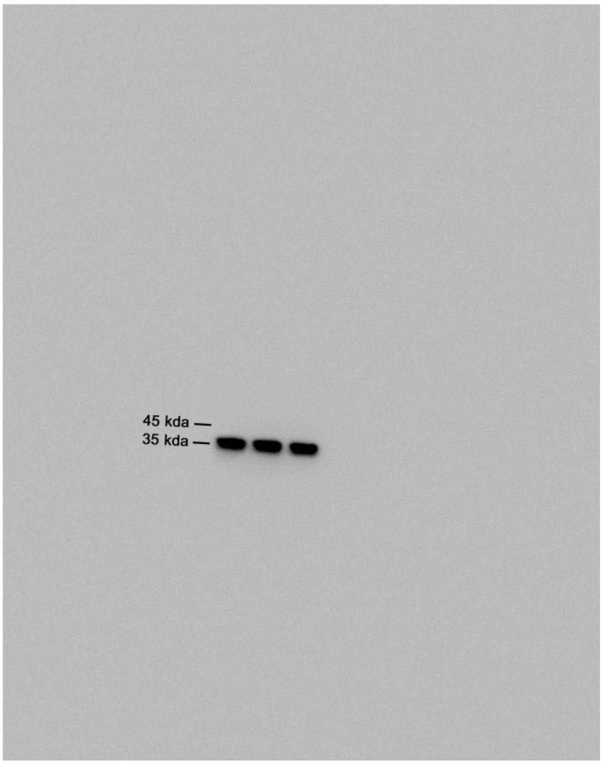

GAPDH

Figure 1h

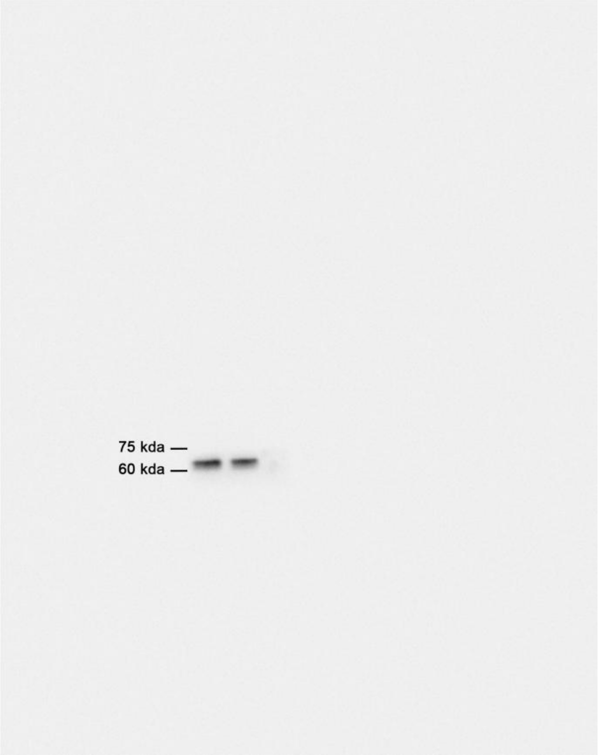

IGF2BP1

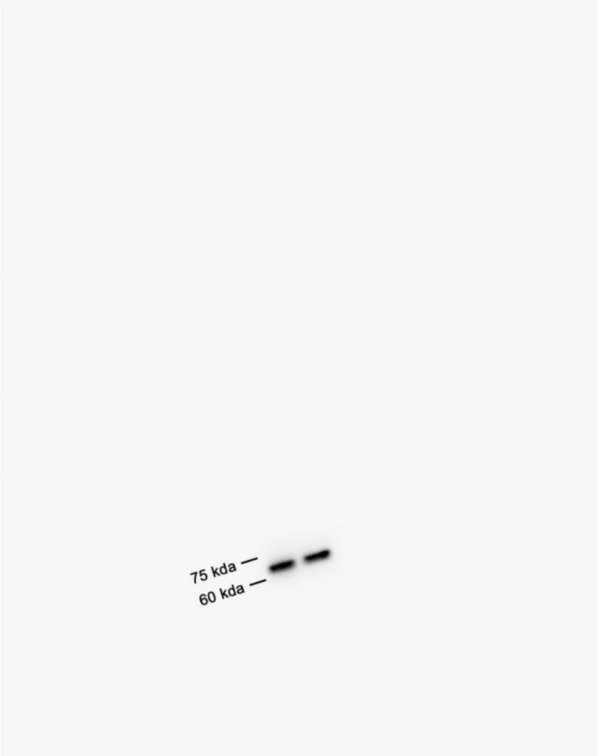

IGF2BP2

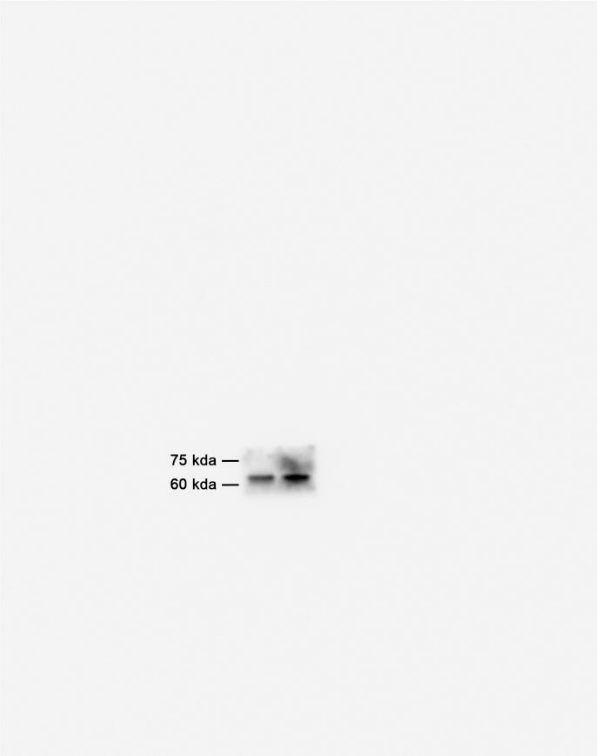

IGF2BP3

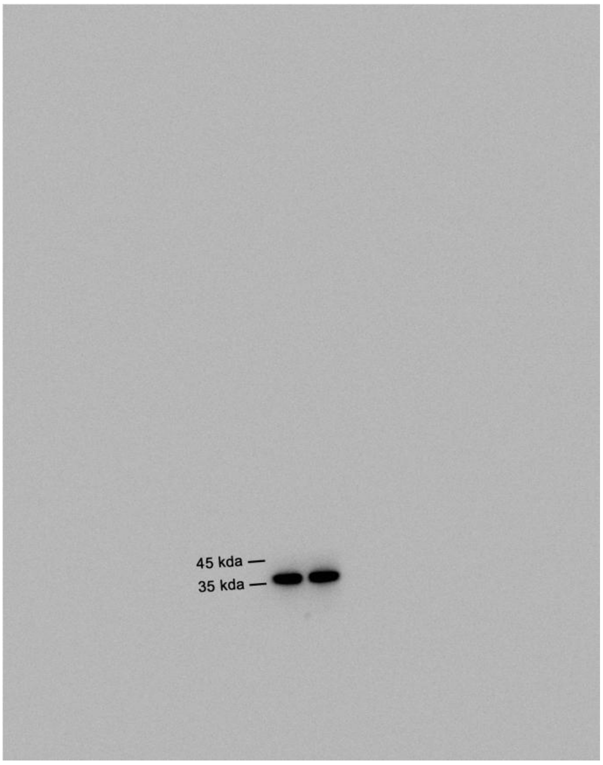

GAPDH

Figure 1k

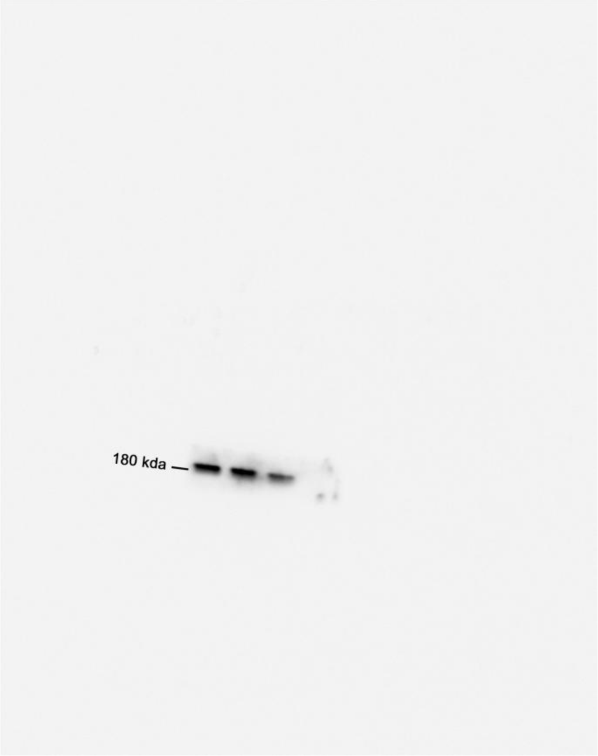

ZO-1

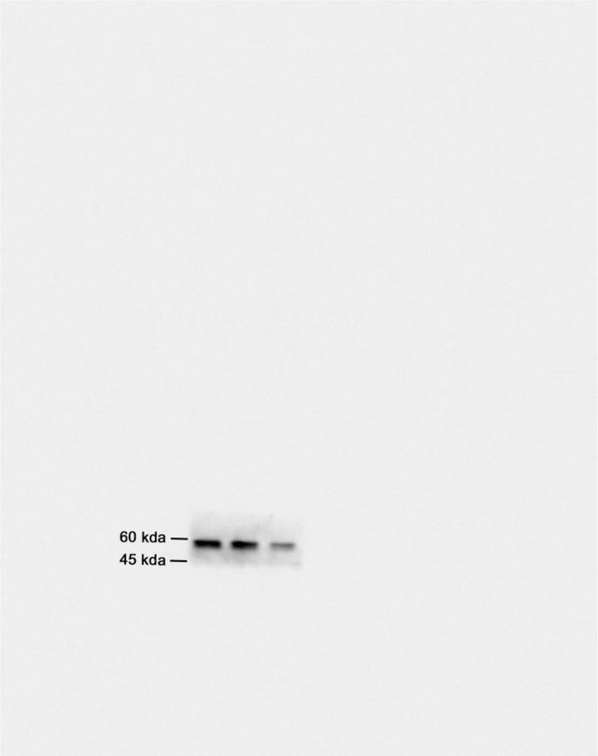

occludin

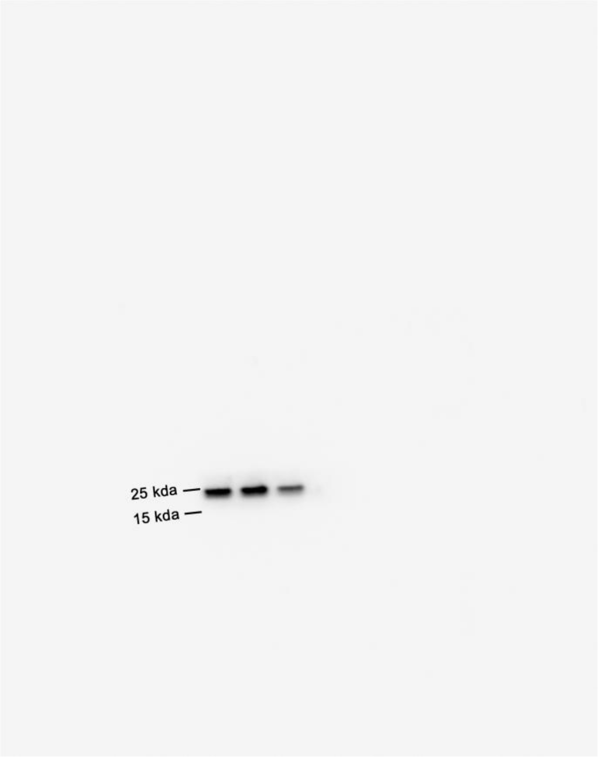

claudin-5

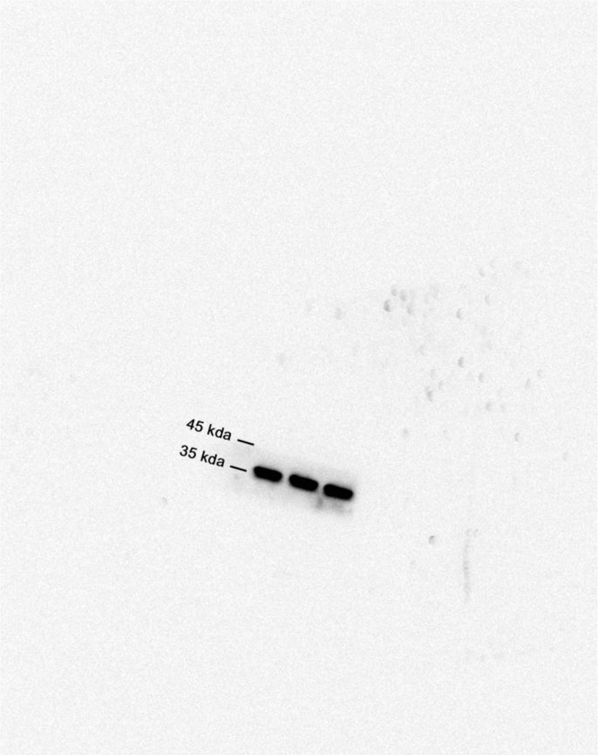

GAPDH

Figure 2h

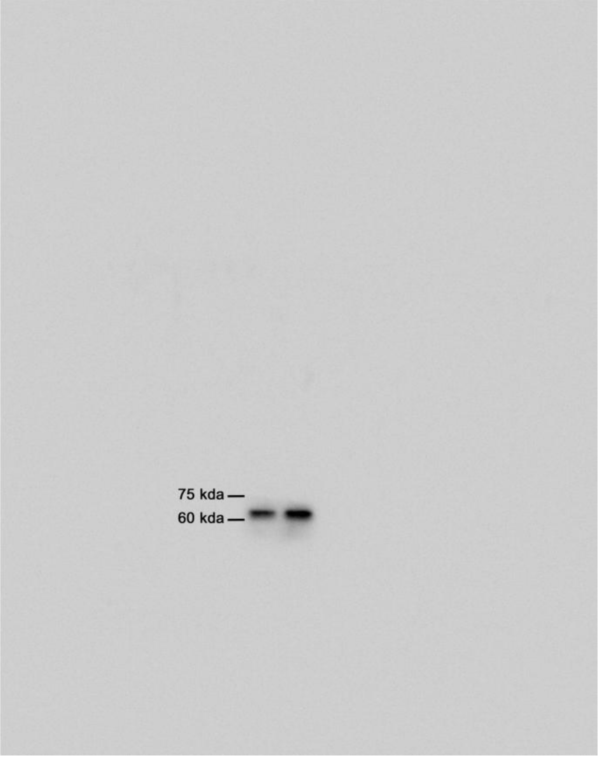

CPEB2

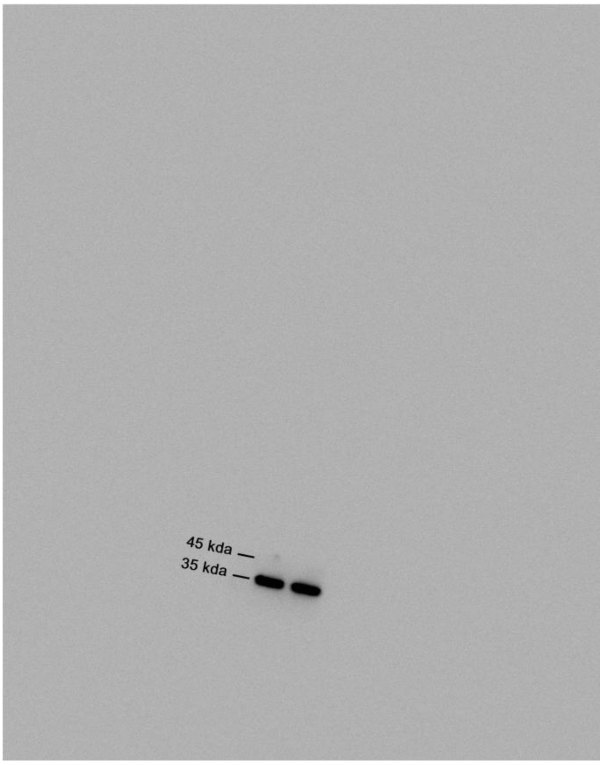

GAPDH

Figure 2k

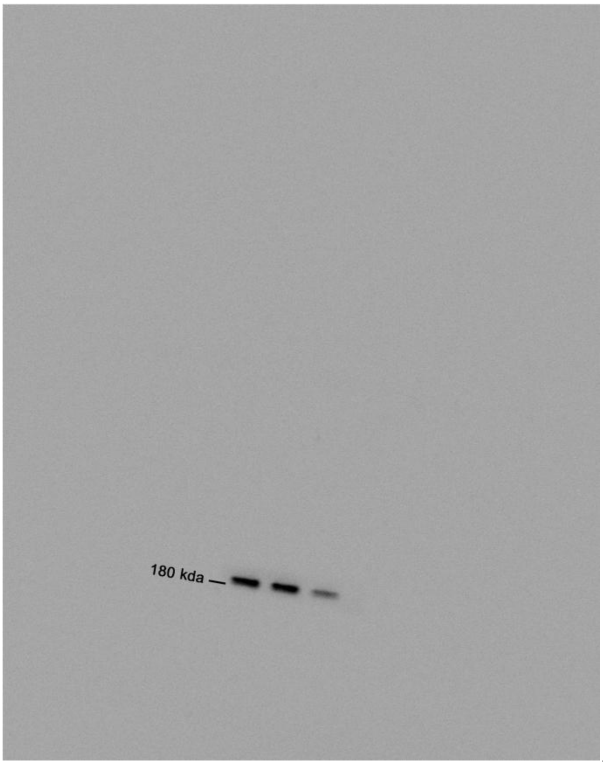

ZO-1

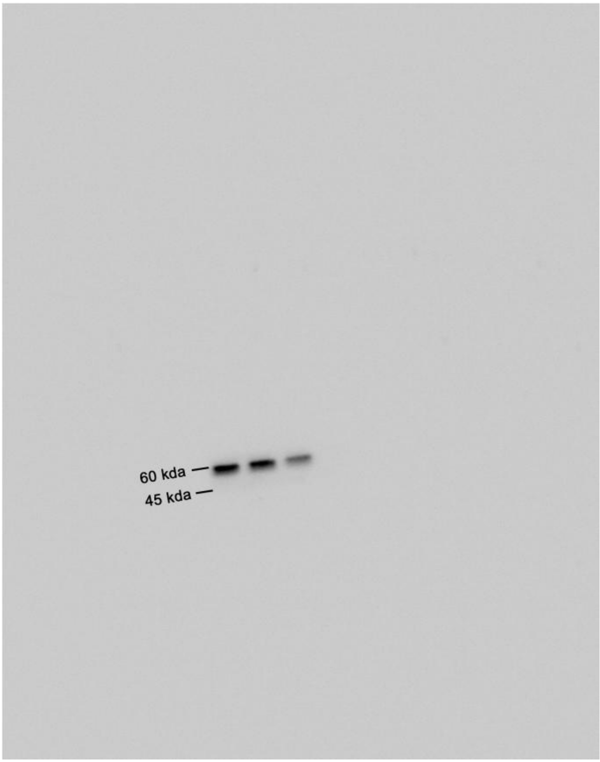

occludin

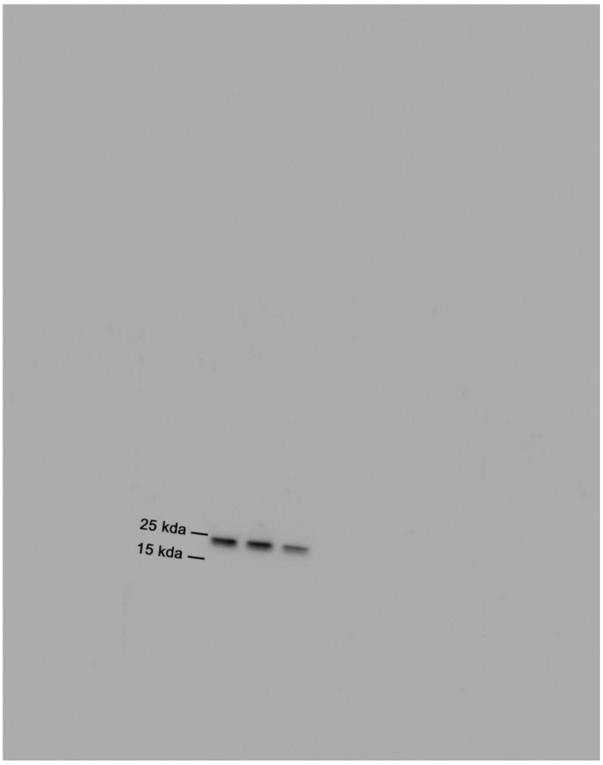

claudin-5

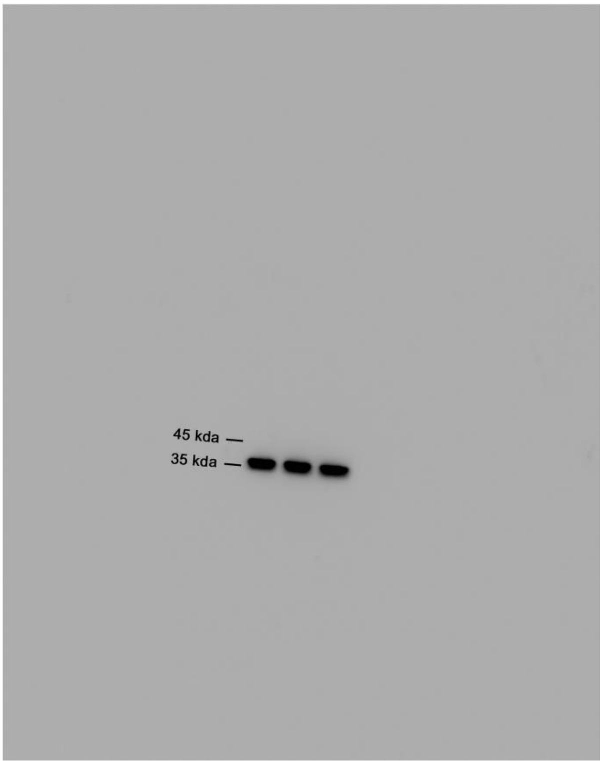

GAPDH

Figure 3n

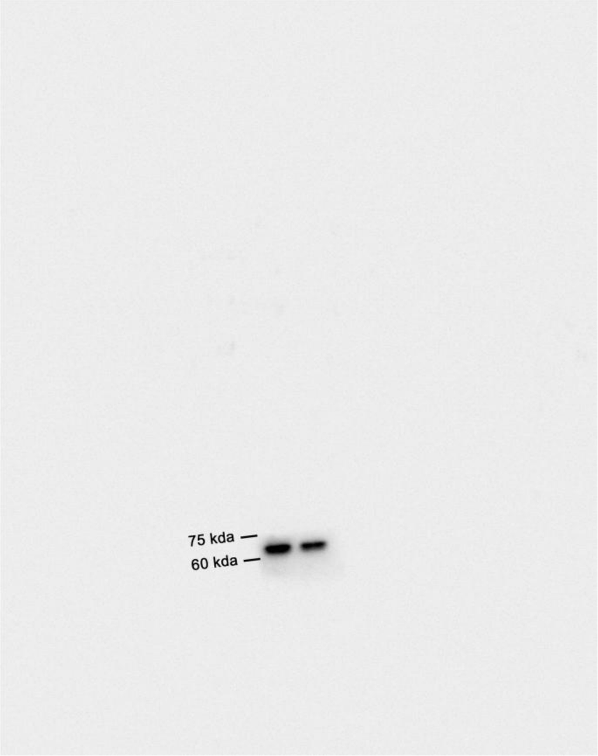

CPEB2

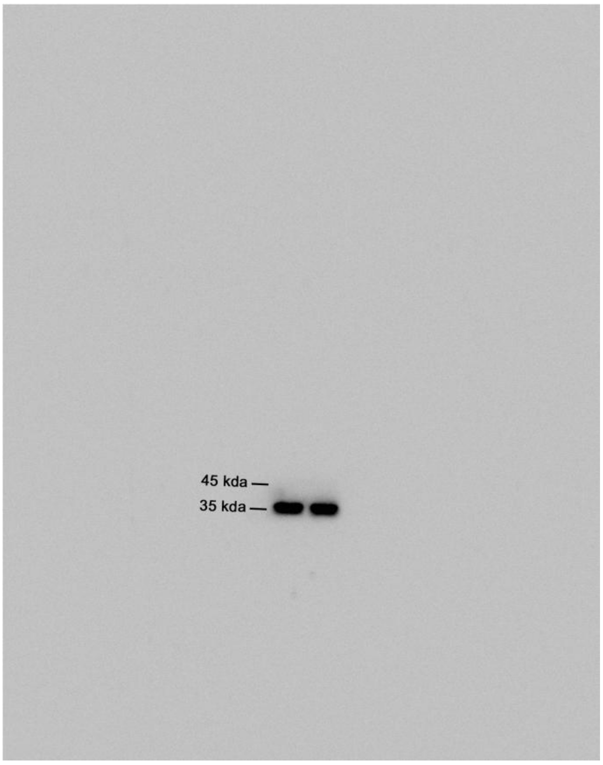

GAPDH

Figure 3q

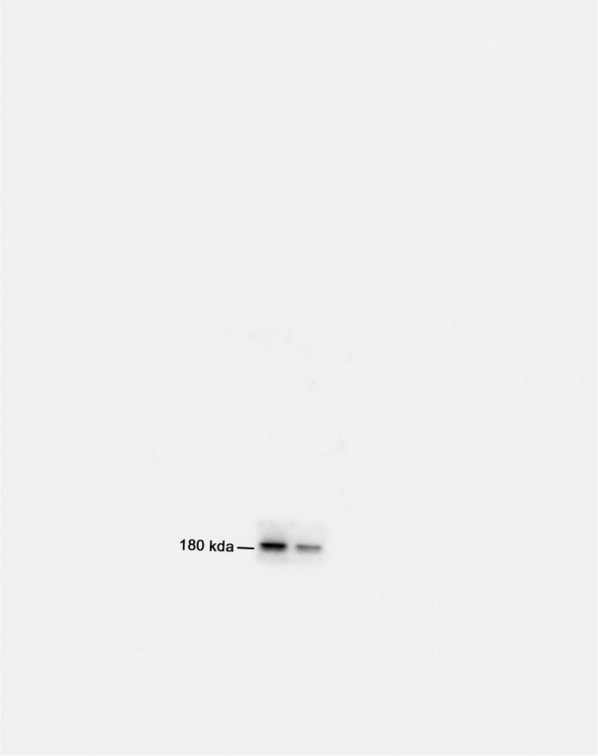

ZO-1

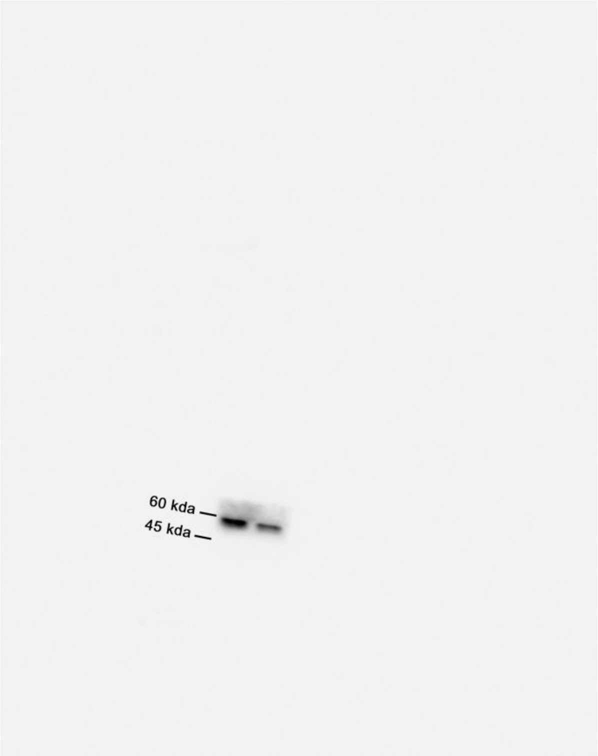

occludin

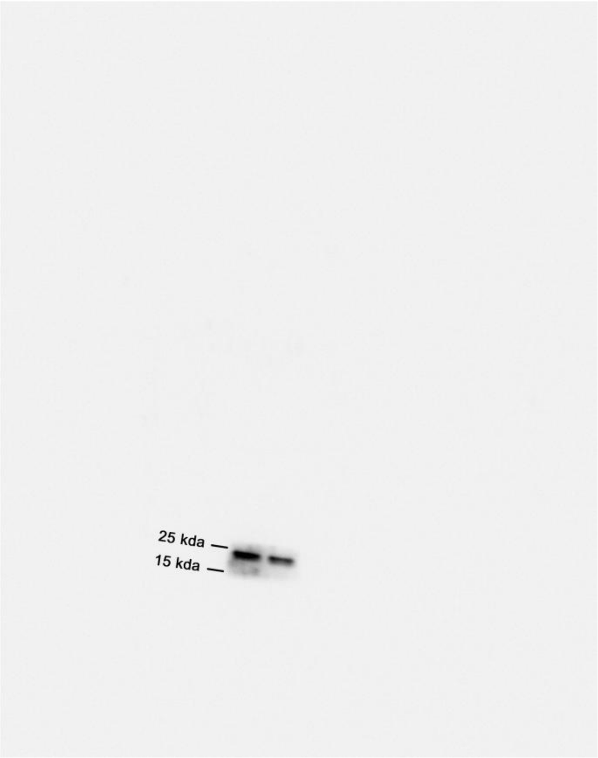

claudin-5

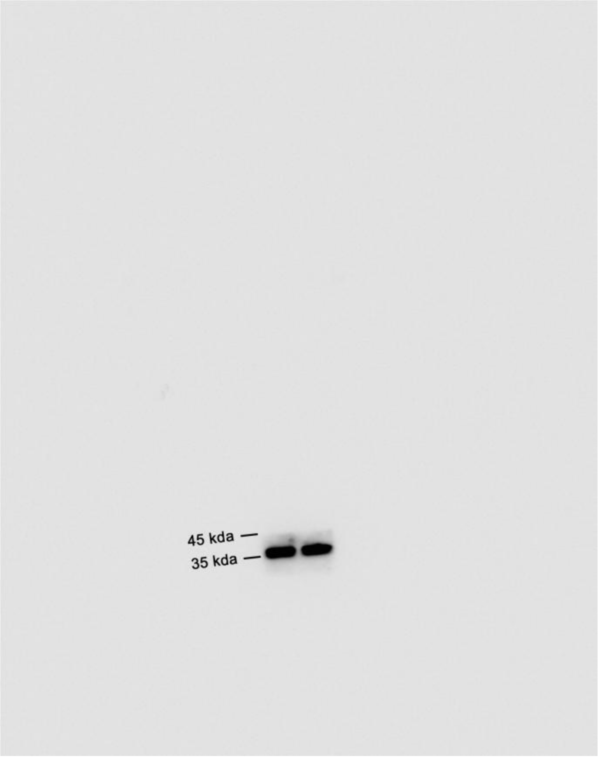

GAPDH

Figure 3u

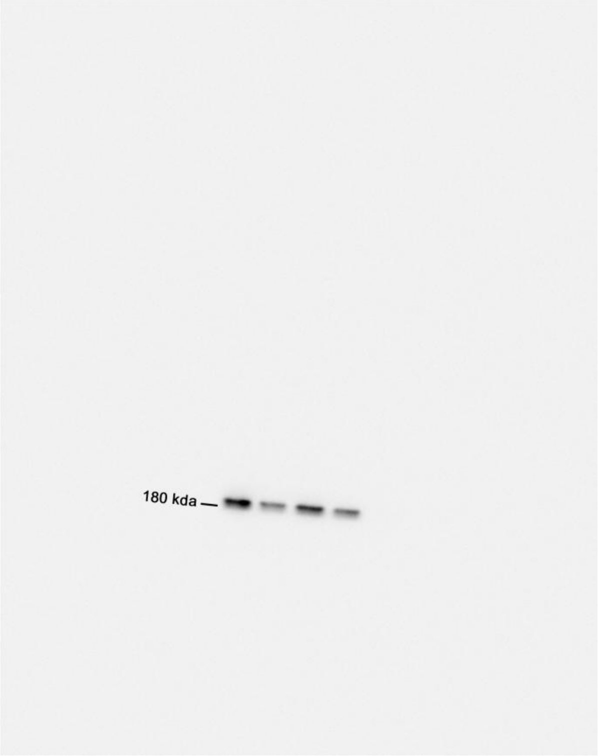

ZO-1

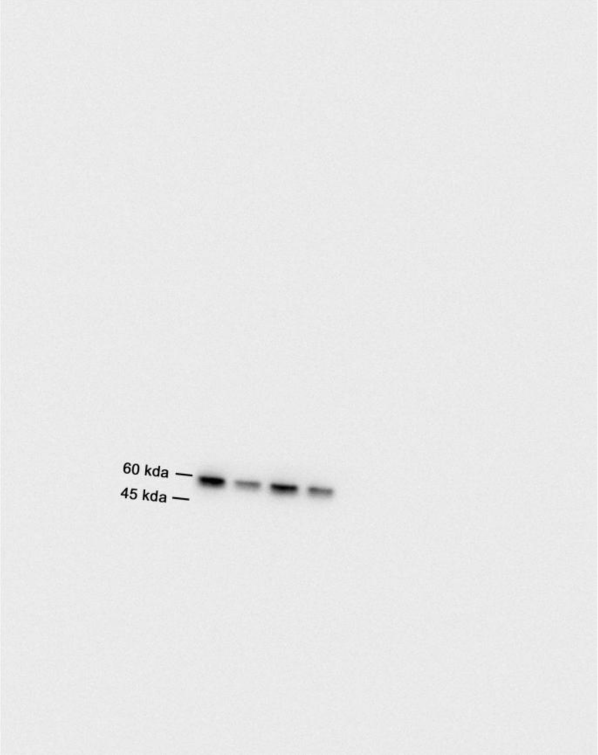

occludin

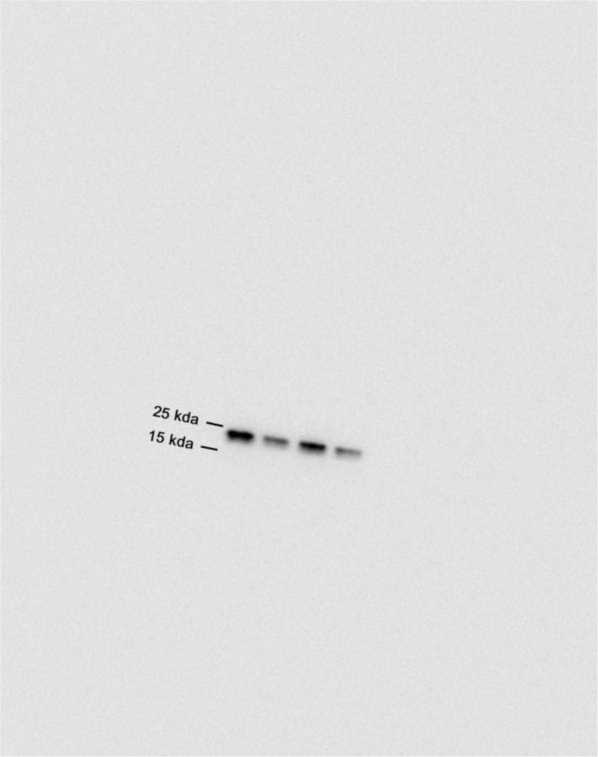

claudin-5

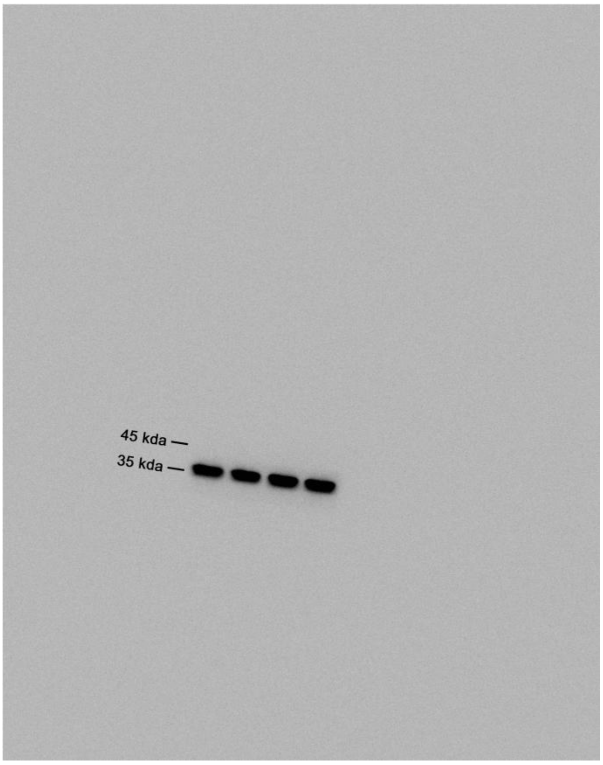

GAPDH

Figure 4e

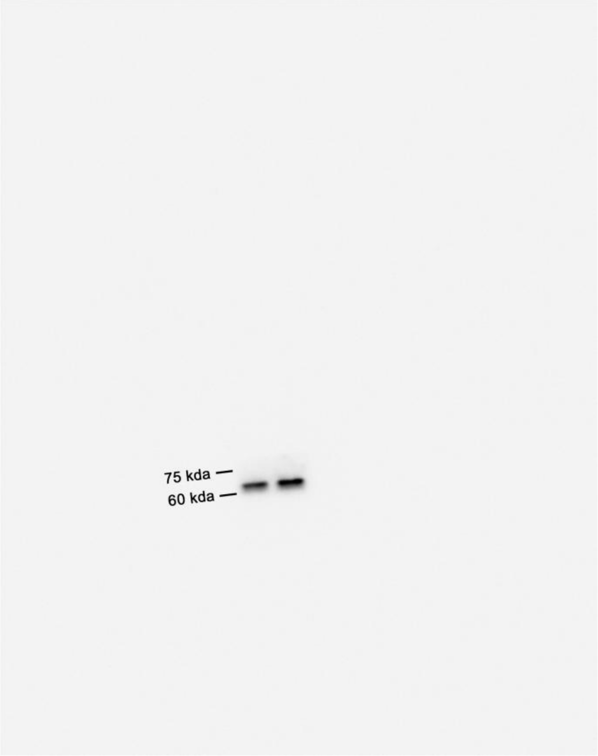

SRSF5

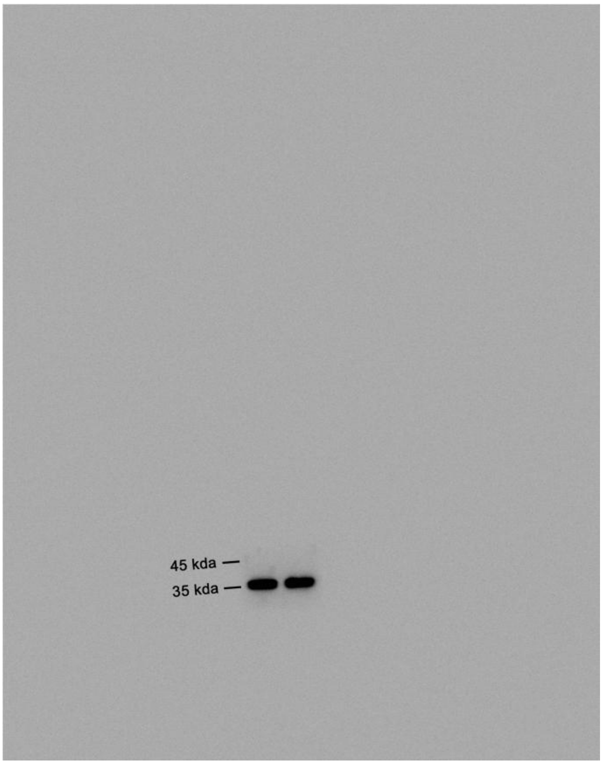

GAPDH

Figure 4h

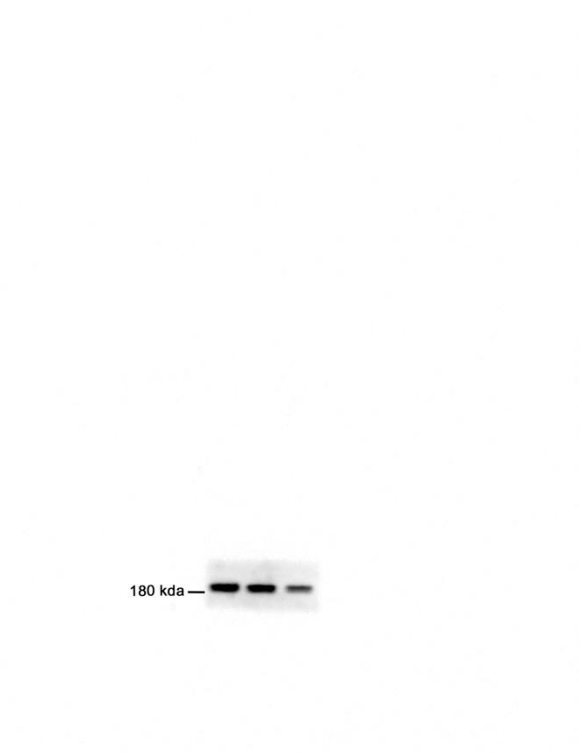

ZO-1

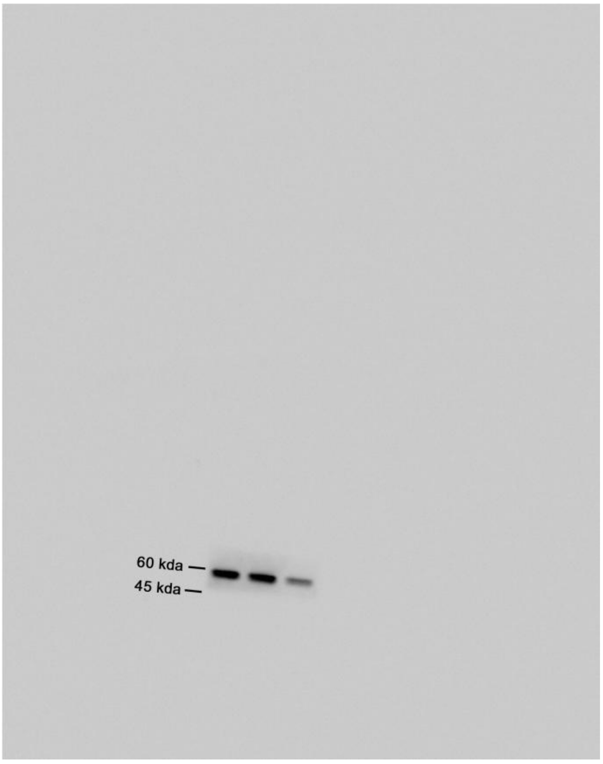

occludin

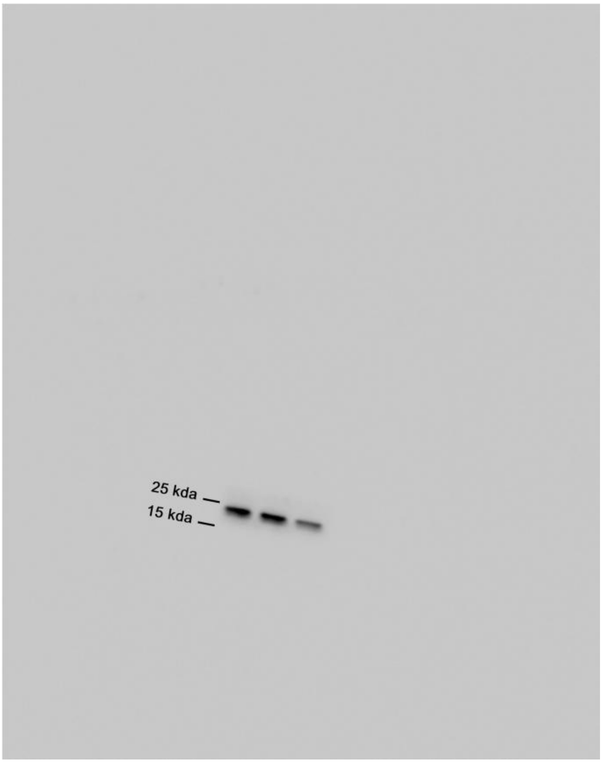

claudin-5

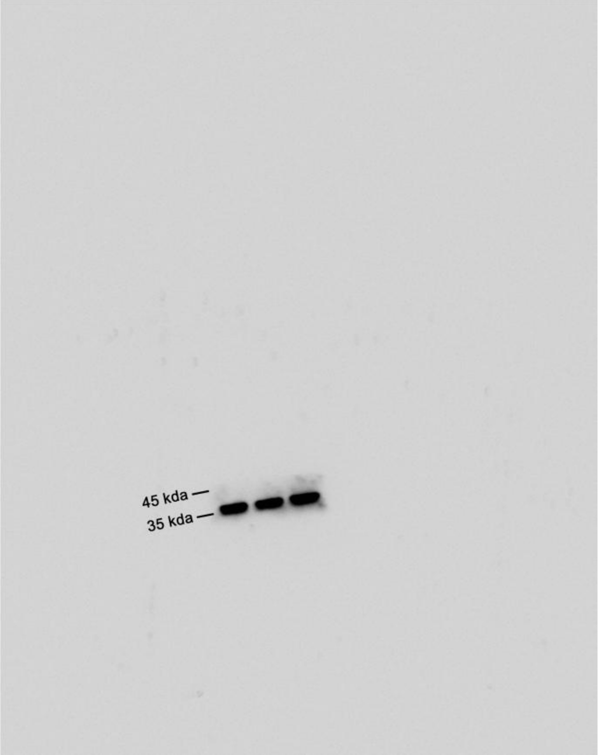

GAPDH

Figure 41

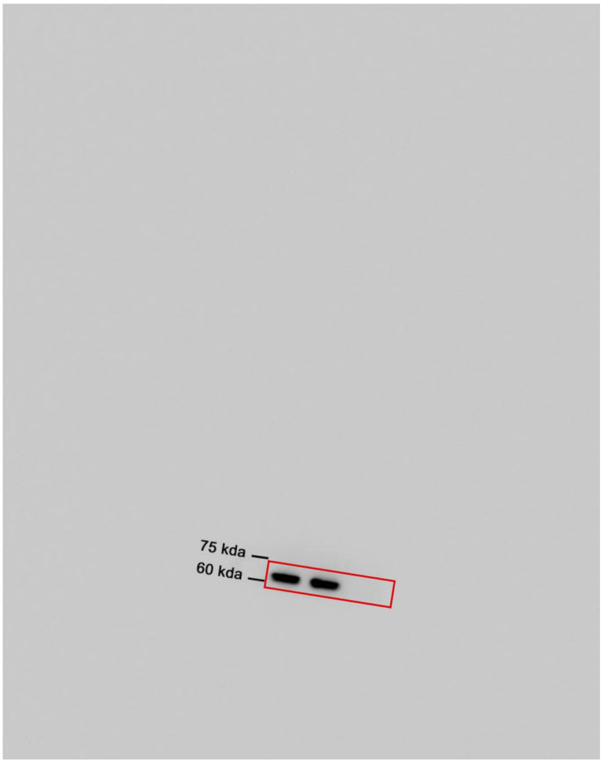

CPEB2

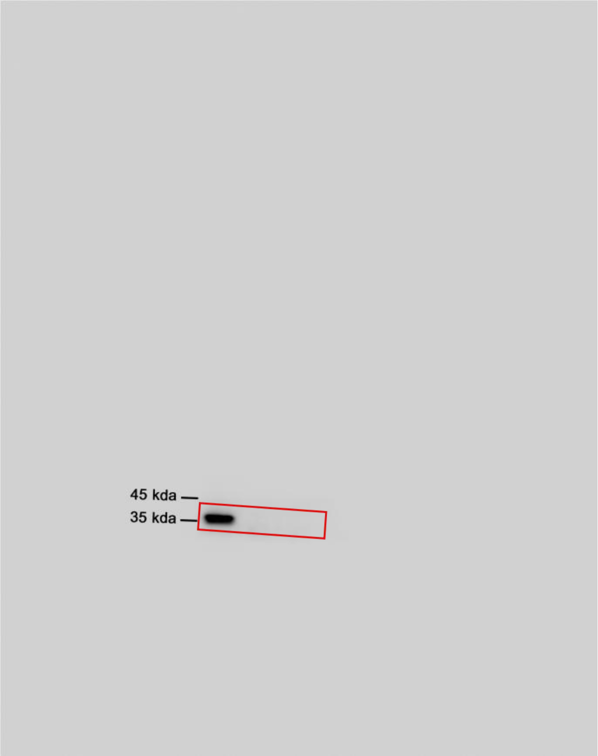

GAPDH

Figure 5a

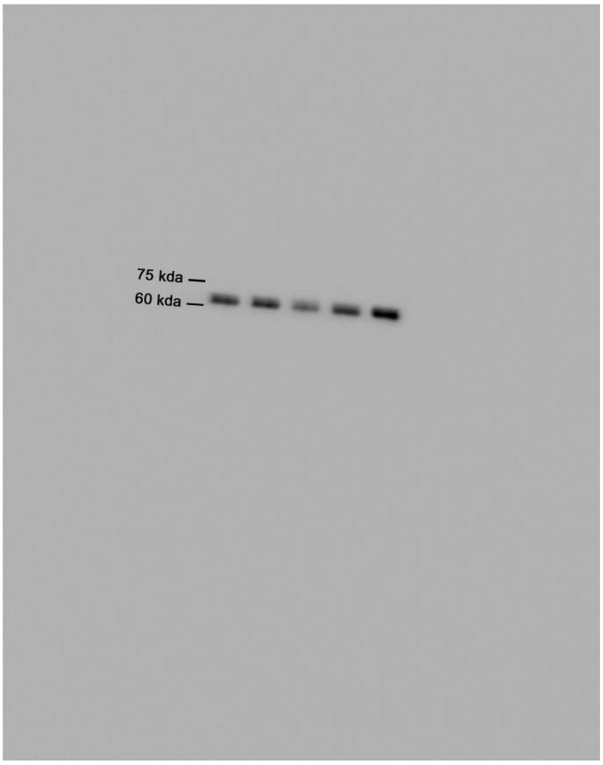

SRSF5

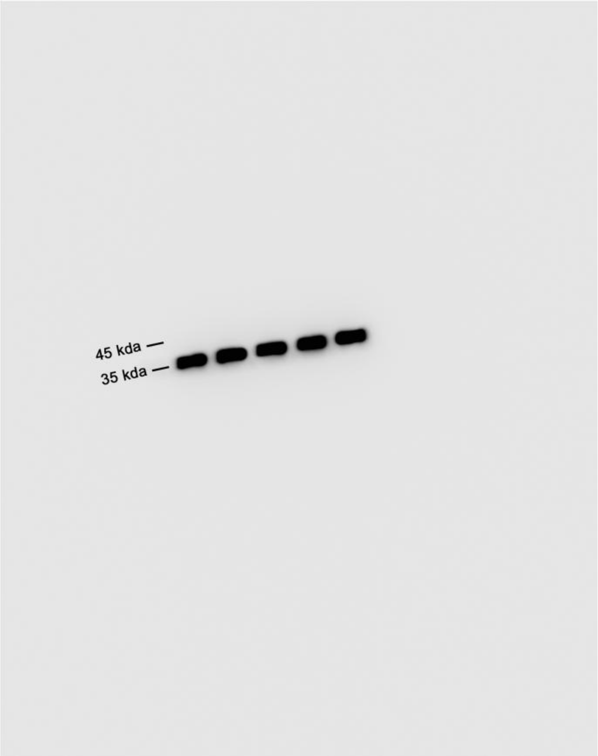

GAPDH

Figure 5d

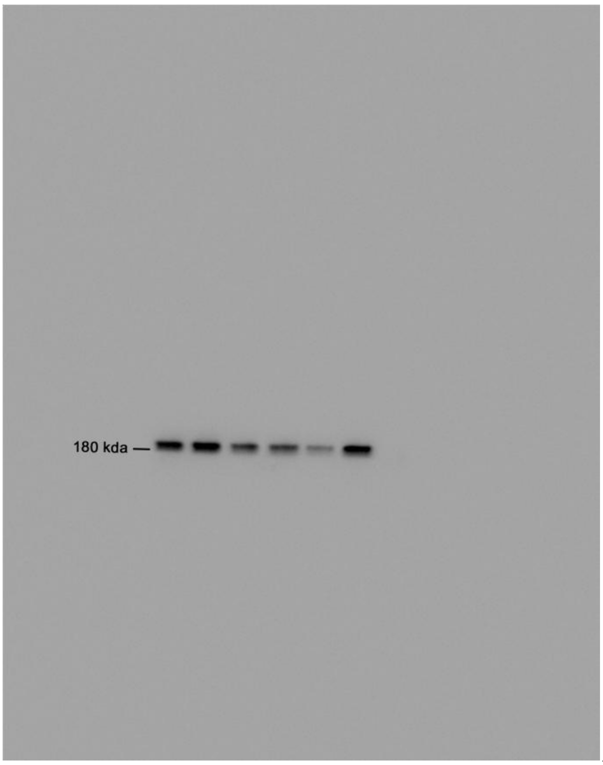

ZO-1

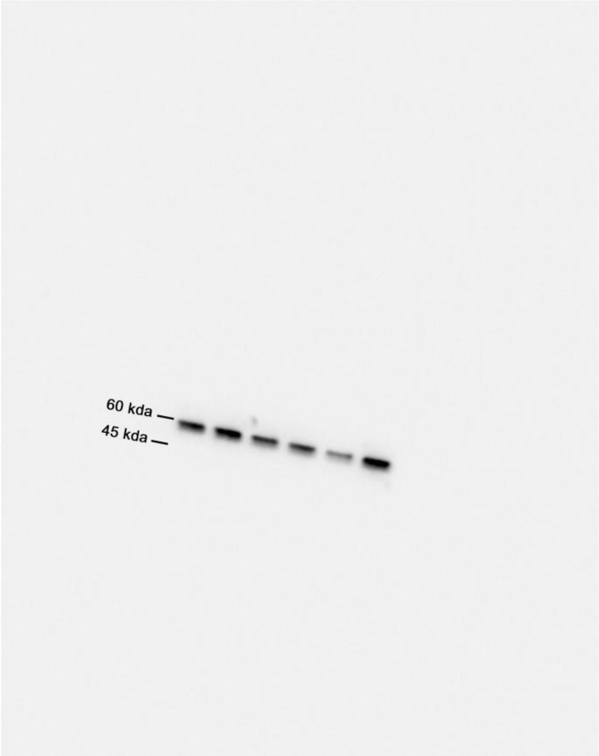

occludin

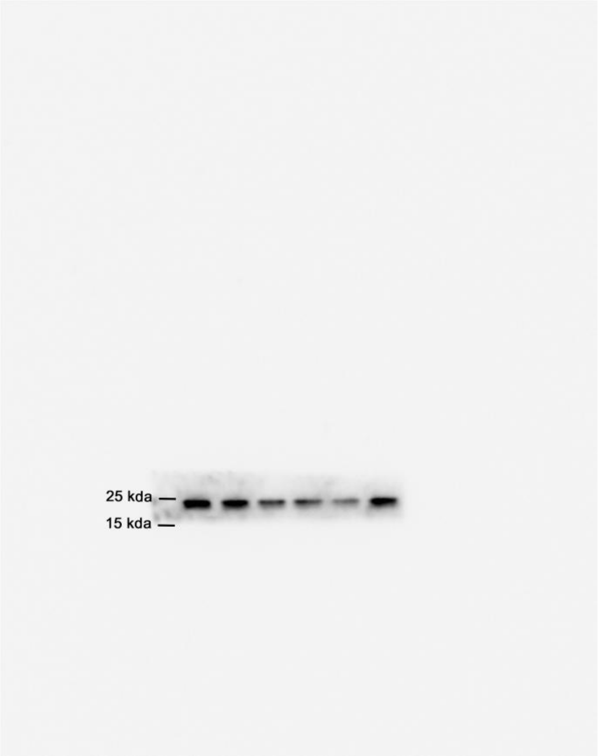

claudin-5

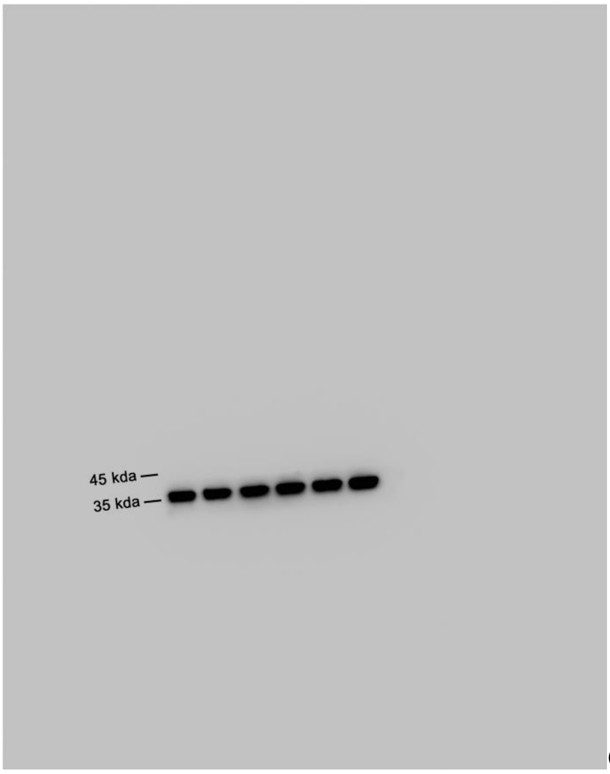

GAPDH

Figure 6f

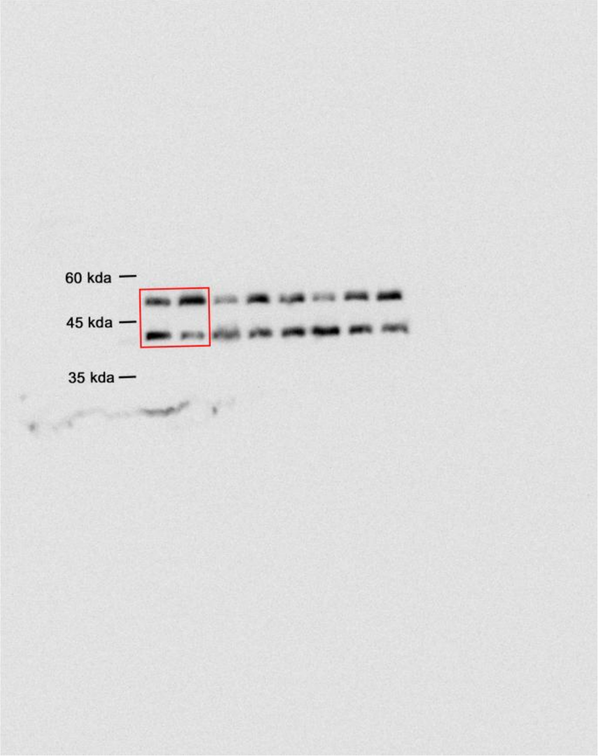

ETS1

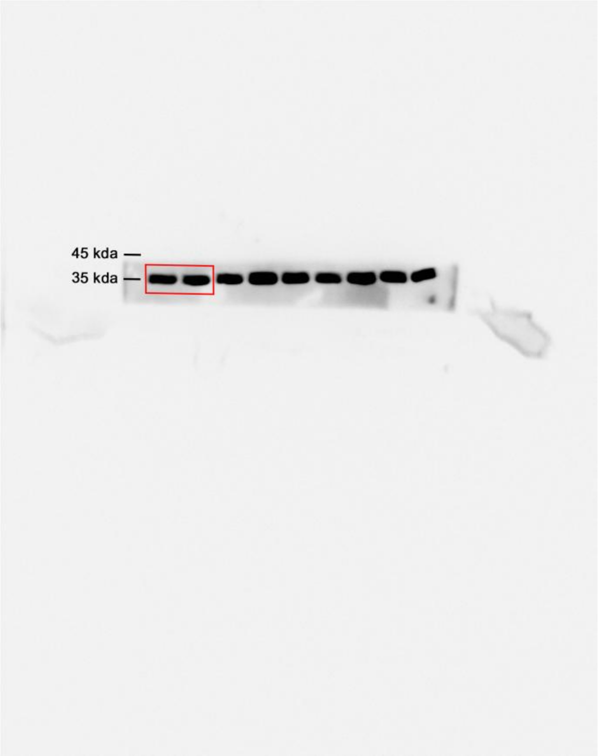

GAPDH

Figure 6m

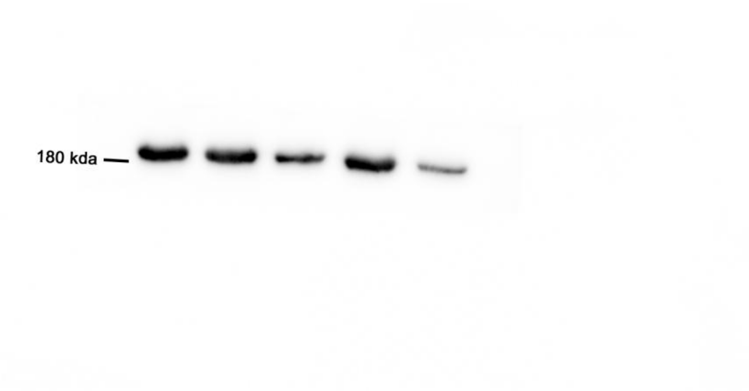

ZO-1

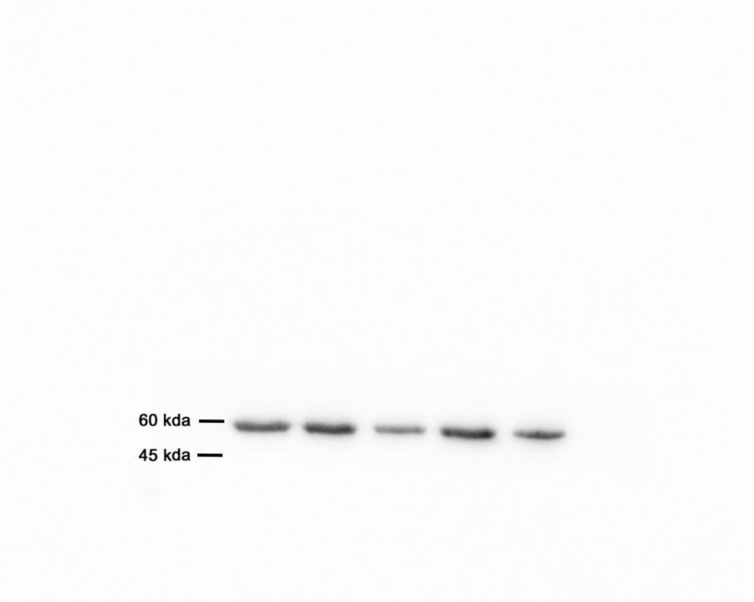

occludin

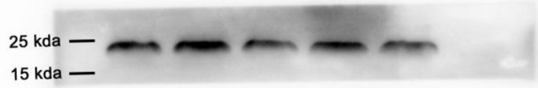

claudin-5

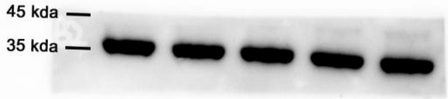

GAPDH

Figure 7c

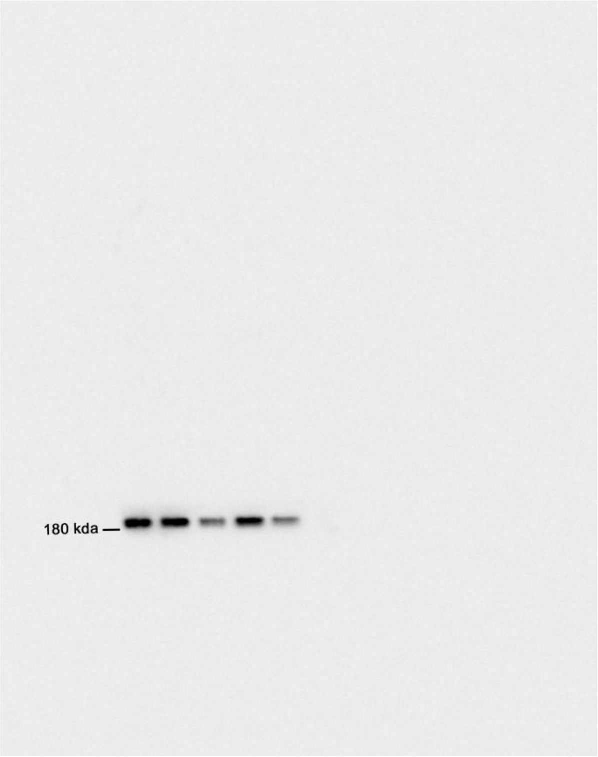

ZO-1

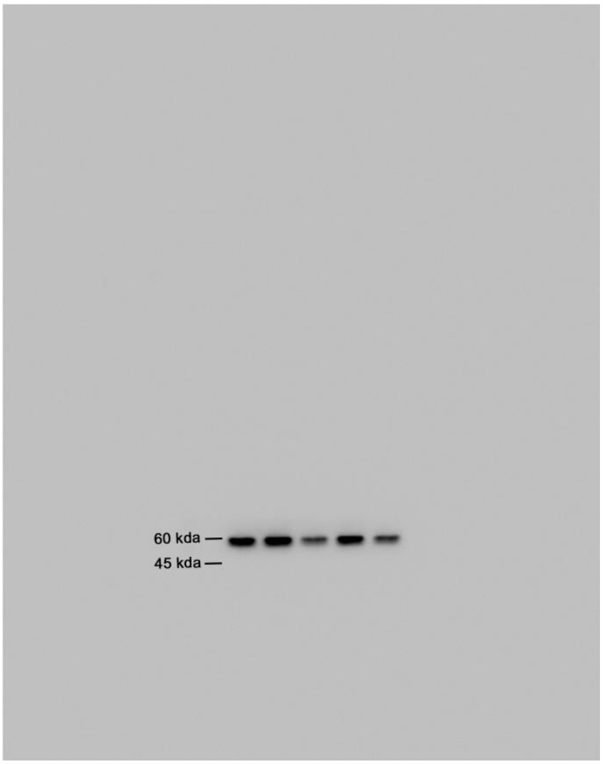

occludin

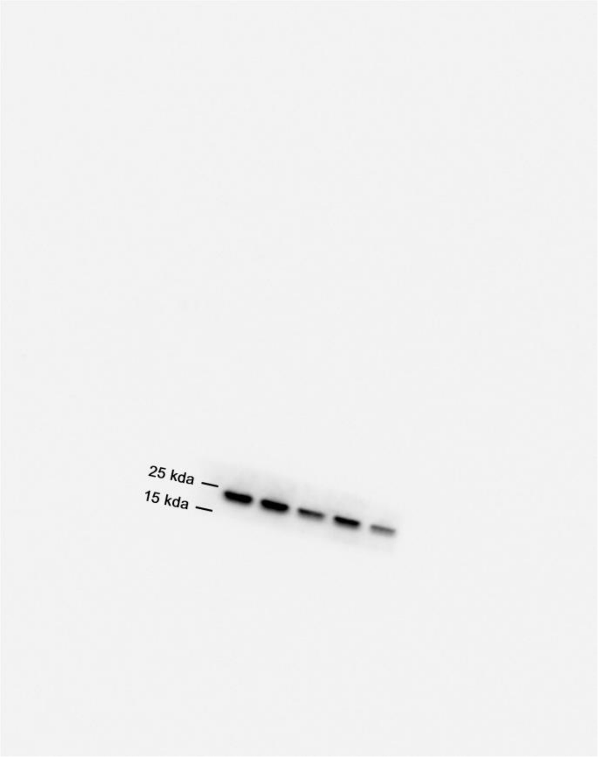

claudin-5

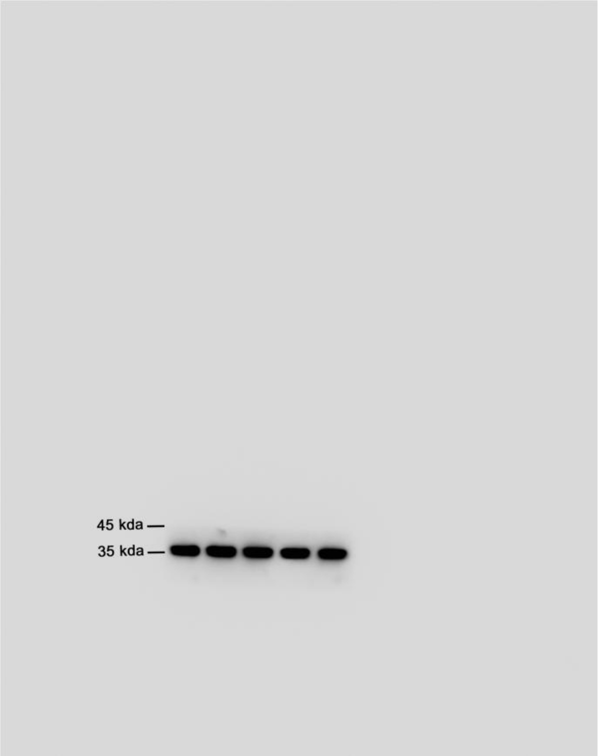

GAPDH

Figure S1b

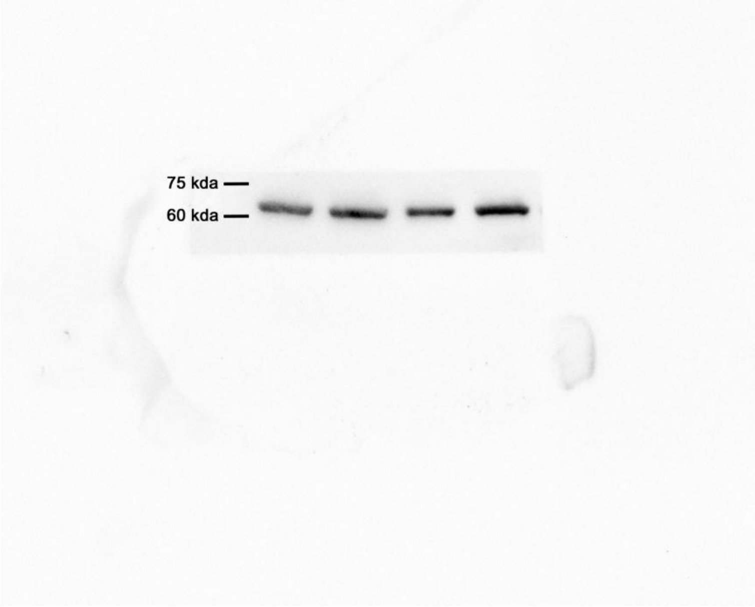

METTL3

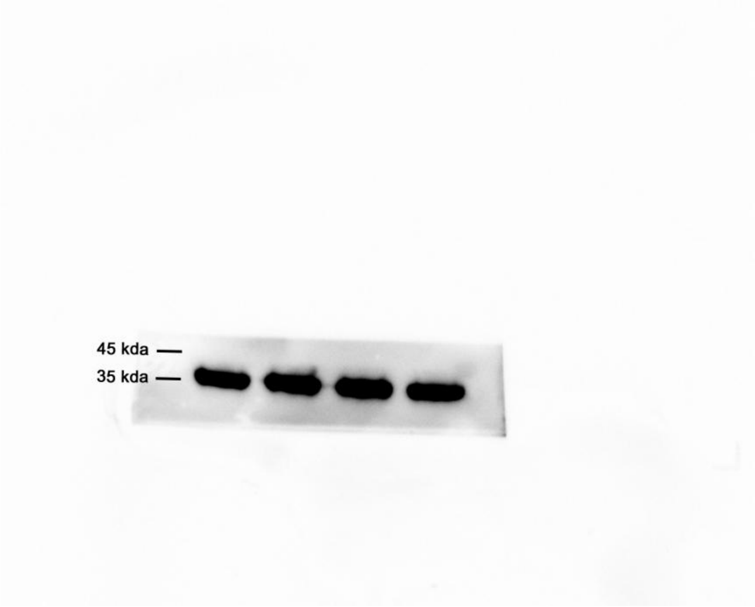

GAPDH

Figure S2a

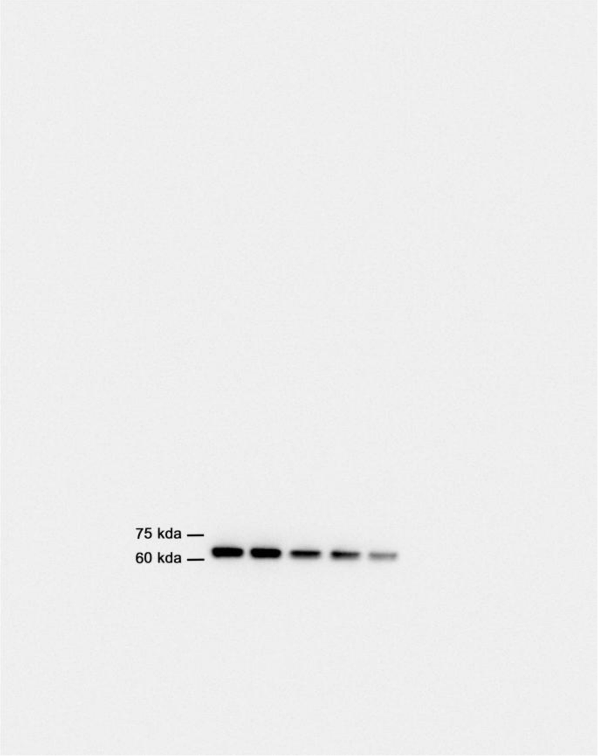

METTL3

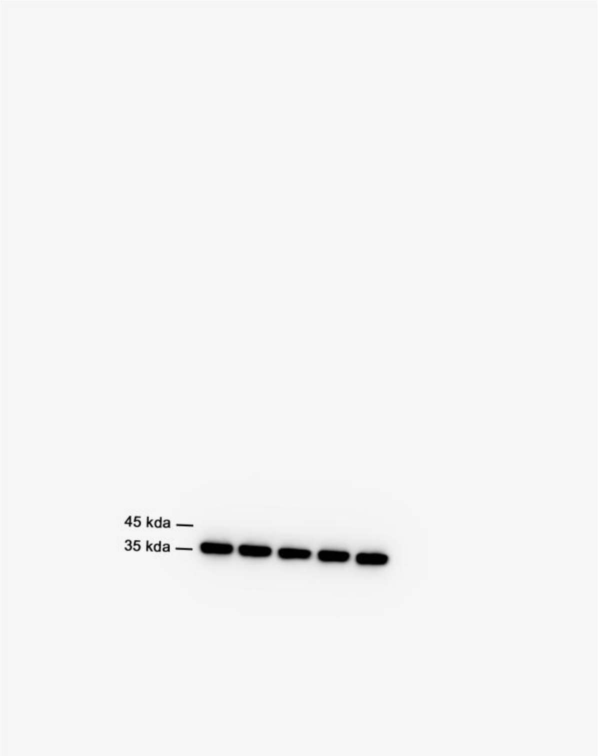

GAPDH

Figure S2b

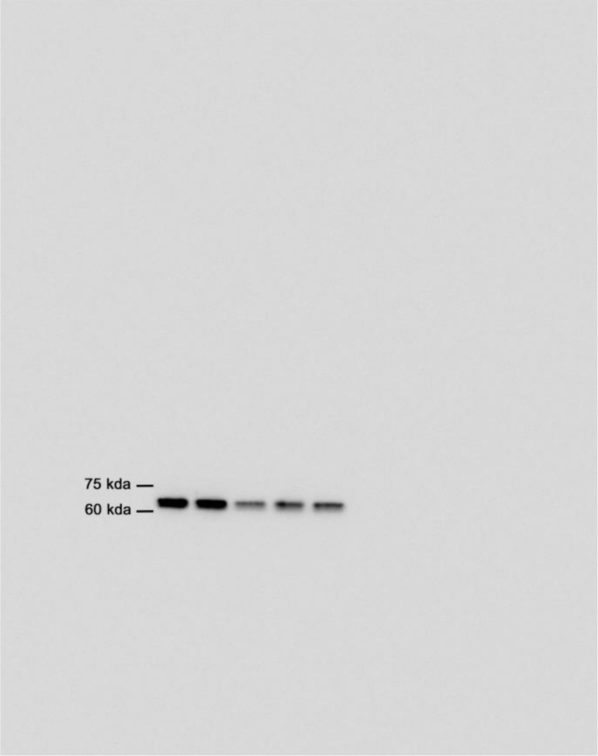

IGF2BP3

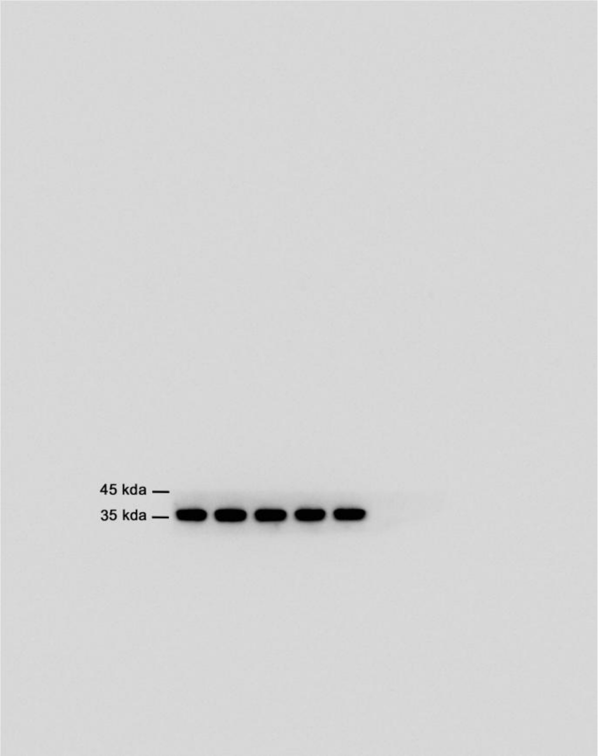

GAPDH

Figure S2c

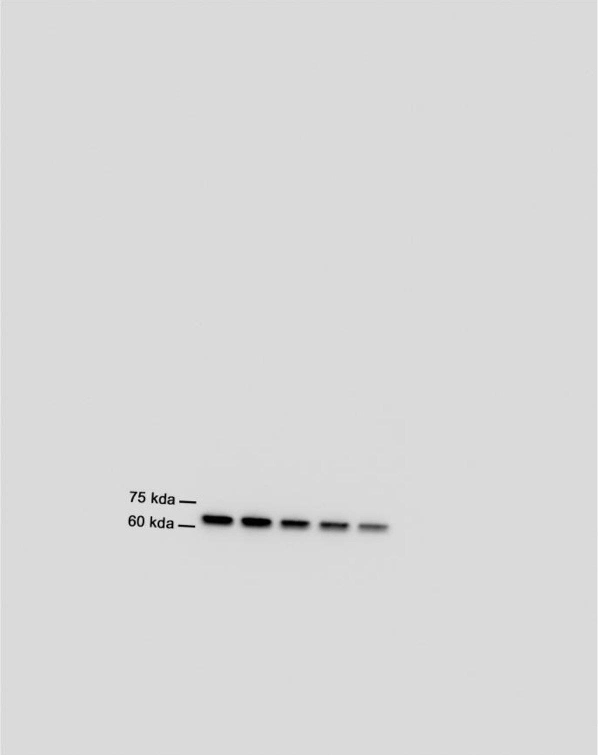

CPEB2

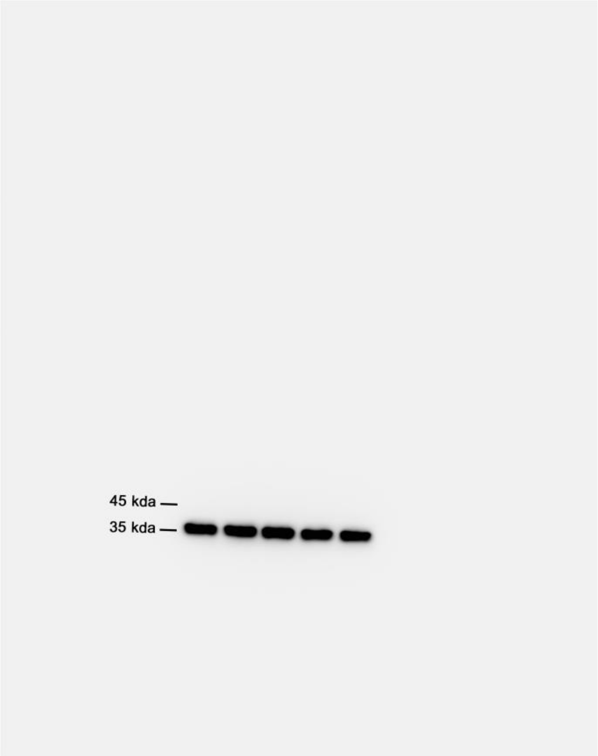

GAPDH

Figure S2d

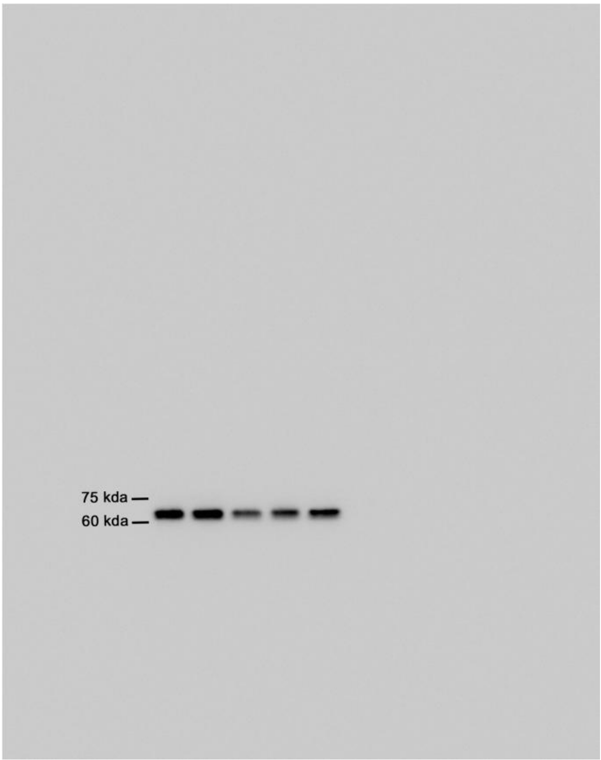

SRSF5

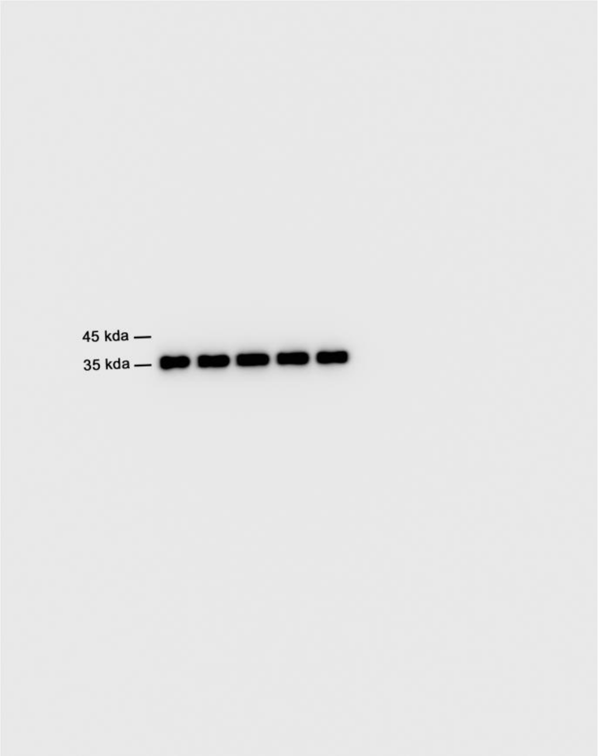

GAPDH

Figure S2e

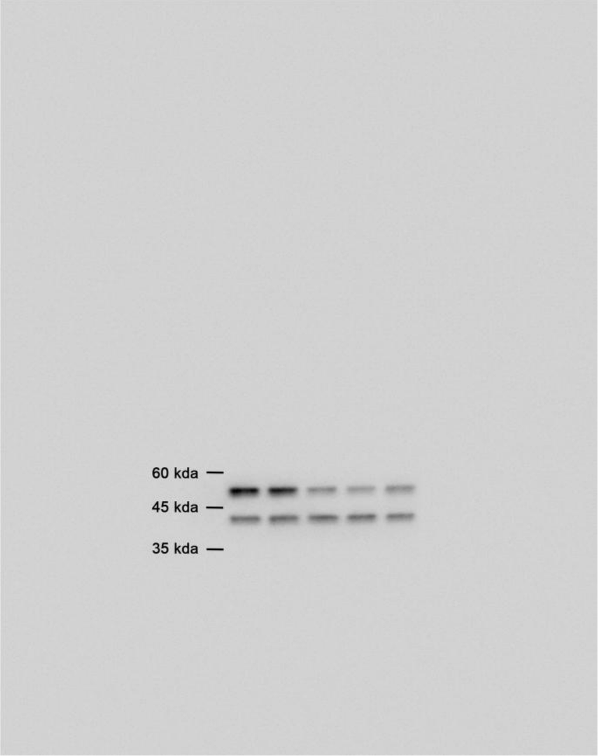

P51-ETS1

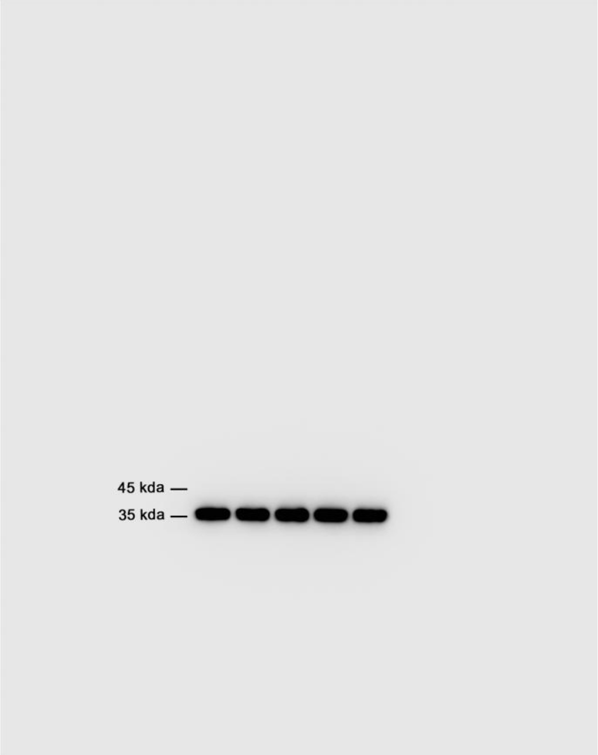

GAPDH
