# Supplementary material for: CPEB2 m6A methylation regulates blood–tumor barrier permeability by regulating splicing factor SRSF5 stability
Source: Commun Biol. 2022 Sep 5;5:908. doi: 10.1038/s42003-022-03878-9 (PMC9445078; doi:10.1038/s42003-022-03878-9)
Supplement: Supplementary file 2 — Description of Additional Supplementary Files [file 42003_2022_3878_MOESM2_ESM.pdf]

Description of Additional Supplementary Files

File name: Supplementary Data 1

Description: The numerical data that make up the all graphs in the paper.
