# Supplementary material for: CPEB2 m6A methylation regulates blood–tumor barrier permeability by regulating splicing factor SRSF5 stability
Source: Commun Biol. 2022 Sep 5;5:908. doi: 10.1038/s42003-022-03878-9 (PMC9445078; doi:10.1038/s42003-022-03878-9)
Supplement: Supplementary file 4 — Reporting Summary [file 42003_2022_3878_MOESM4_ESM.pdf]

nature portfolio

Corresponding author(s): DBPR COMMSBIO-22-0546B

Last updated by author(s): Aug 3, 2022

Reporting Summary

Nature Portfolio wishes to improve the reproducibility of the work that we publish. This form provides structure for consistency and transparency in reporting. For further information on Nature Portfolio policies, see our Editorial Policies and the Editorial Policy Checklist.

Statistics

For all statistical analyses, confirm that the following items are present in the figure legend, table legend, main text, or Methods section.

n/a	Confirmed	
☐	☒	The exact sample size (*n*) for each experimental group/condition, given as a discrete number and unit of measurement
☒	☐	A statement on whether measurements were taken from distinct samples or whether the same sample was measured repeatedly
☐	☒	The statistical test(s) used AND whether they are one- or two-sided<br>*Only common tests should be described solely by name; describe more complex techniques in the Methods section.*
☒	☐	A description of all covariates tested
☒	☐	A description of any assumptions or corrections, such as tests of normality and adjustment for multiple comparisons
☐	☒	A full description of the statistical parameters including central tendency (e.g. means) or other basic estimates (e.g. regression coefficient) AND variation (e.g. standard deviation) or associated estimates of uncertainty (e.g. confidence intervals)
☐	☒	For null hypothesis testing, the test statistic (e.g. *F*, *t*, *r*) with confidence intervals, effect sizes, degrees of freedom and *P* value noted<br>*Give P values as exact values whenever suitable.*
☒	☐	For Bayesian analysis, information on the choice of priors and Markov chain Monte Carlo settings
☒	☐	For hierarchical and complex designs, identification of the appropriate level for tests and full reporting of outcomes
☒	☐	Estimates of effect sizes (e.g. Cohen's *d*, Pearson's *r*), indicating how they were calculated

Our web collection on statistics for biologists contains articles on many of the points above.

Software and code

Policy information about availability of computer code

Data collection	no software was used
Data analysis	no software was used

For manuscripts utilizing custom algorithms or software that are central to the research but not yet described in published literature, software must be made available to editors and reviewers. We strongly encourage code deposition in a community repository (e.g. GitHub). See the Nature Portfolio guidelines for submitting code & software for further information.

Data

Policy information about availability of data

All manuscripts must include a data availability statement. This statement should provide the following information, where applicable:

- Accession codes, unique identifiers, or web links for publicly available datasets
- A description of any restrictions on data availability
- For clinical datasets or third party data, please ensure that the statement adheres to our policy

All data that support the findings of this study are available from the researchers on reasonable request.

Human research participants

Policy information about studies involving human research participants and Sex and Gender in Research.

Reporting on sex and gender	N/A
Population characteristics	N/A
Recruitment	N/A
Ethics oversight	N/A

Note that full information on the approval of the study protocol must also be provided in the manuscript.

Field-specific reporting

Please select the one below that is the best fit for your research. If you are not sure, read the appropriate sections before making your selection.

☒ Life sciences ☐ Behavioural & social sciences ☐ Ecological, evolutionary & environmental sciences

For a reference copy of the document with all sections, see nature.com/documents/nr-reporting-summary-flat.pdf

Life sciences study design

All studies must disclose on these points even when the disclosure is negative.

Sample size	All experiments were performed at least in triplicate. Statistical analysis was performed using Student's t test and one-way analysis of variance in GraphPad Prism software (v5.01; GraphPad Software, La Jolla, CA, USA), and data are presented as the mean ± standard deviation. A $P < 0.05$ was considered significant.
Data exclusions	No data were excluded from the analyses.
Replication	Findings were reliably reproduced according to the methods.
Randomization	Samples were allocated into experimental groups randomly.
Blinding	N/A

Reporting for specific materials, systems and methods

We require information from authors about some types of materials, experimental systems and methods used in many studies. Here, indicate whether each material, system or method listed is relevant to your study. If you are not sure if a list item applies to your research, read the appropriate section before selecting a response.

Materials & experimental systems

n/a	Involved in the study	
☐	☒	Antibodies
☐	☒	Eukaryotic cell lines
☒	☐	Palaeontology and archaeology
☐	☒	Animals and other organisms
☒	☐	Clinical data
☒	☐	Dual use research of concern

Methods

n/a	Involved in the study	
☒	☐	ChIP-seq
☒	☐	Flow cytometry
☒	☐	MRI-based neuroimaging

Antibodies

Antibodies used

METTL3 15073-1-AP, Proteintech
IGF2BP1 22803-1-AP, Proteintech
IGF2BP2 11601-1-AP, Proteintech
IGF2BP3 14642-1-AP, Proteintech
CPEB2 ab51069, Abcam
SRSF5 ab67175, Abcam

P51-ETS1 sc-55581,Santa Cruz
P42-ETS1 sc-55581,Santa Cruz
GAPDH 60004-1-lg, Proteintech
IgG ab18413, Abcam
ZO-1 61-7300, Thermo Fisher
Occludin 71-1500, Thermo Fisher
Claudin-5 35-2500, Thermo Fisher
Goat anti-mouse SA00001-1, Proteintech
Goat anti-rabbit SA00001-2, Proteintech

Validation	All antibodys were KD/KO validated.

Eukaryotic cell lines

Policy information about cell lines and Sex and Gender in Research

Cell line source(s)	The hCMEC/D3 cells (ECs) immortalized human brain endothelial cell line was provided by Dr. Couraud from the Cochin Institute in Paris, France. The U251 human glioblastoma and HEK293T cell lines were purchased from the Cell Resource Center of Shanghai Institute of Biological Sciences. Normal human astrocytes (NHA) were purchased from ScienCell Research Laboratories (Carlsbad, CA, USA).
Authentication	All cell lines have STR authentication.
Mycoplasma contamination	All cell lines tested negative for mycoplasma contamination.
Commonly misidentified lines (See ICLAC register)	N/A

Animals and other research organisms

Policy information about studies involving animals; ARRIVE guidelines recommended for reporting animal research, and Sex and Gender in Research

Laboratory animals	Female 8-week-old BALB/c nude mice.
Wild animals	N/A
Reporting on sex	N/A
Field-collected samples	N/A
Ethics oversight	All experiments were carried out strictly following the Animal Welfare Act and approved by the Ethics Committee of China Medical University.

Note that full information on the approval of the study protocol must also be provided in the manuscript.
